# Macrophages protect *Talaromyces marneffei* conidia from myeloperoxidase-dependent neutrophil fungicidal activity during infection establishment *in vivo*

**DOI:** 10.1371/journal.ppat.1007063

**Published:** 2018-06-08

**Authors:** Felix Ellett, Vahid Pazhakh, Luke Pase, Erica L. Benard, Harshini Weerasinghe, Denis Azabdaftari, Sultan Alasmari, Alex Andrianopoulos, Graham J. Lieschke

**Affiliations:** 1 Australian Regenerative Medicine Institute, Monash University, Clayton, Victoria, Australia; 2 Cancer and Haematology Division, Walter and Eliza Hall Institute of Medical Research, Parkville, Victoria, Australia; 3 Department of Medical Biology, University of Melbourne, Parkville, Victoria, Australia; 4 Genetics, Genomics and Systems Biology, School of BioSciences, University of Melbourne, Victoria, Australia; Leibniz Institute for Natural Product Research and Infection Biology, GERMANY

## Abstract

Neutrophils and macrophages provide the first line of cellular defence against pathogens once physical barriers are breached, but can play very different roles for each specific pathogen. This is particularly so for fungal pathogens, which can occupy several niches in the host. We developed an infection model of talaromycosis in zebrafish embryos with the thermally-dimorphic intracellular fungal pathogen *Talaromyces marneffei* and used it to define different roles of neutrophils and macrophages in infection establishment. This system models opportunistic human infection prevalent in HIV-infected patients, as zebrafish embryos have intact innate immunity but, like HIV-infected talaromycosis patients, lack a functional adaptive immune system. Importantly, this new talaromycosis model permits thermal shifts not possible in mammalian models, which we show does not significantly impact on leukocyte migration, phagocytosis and function in an established *Aspergillus fumigatus* model. Furthermore, the optical transparency of zebrafish embryos facilitates imaging of leukocyte/pathogen interactions *in vivo*. Following parenteral inoculation, *T*. *marneffei* conidia were phagocytosed by both neutrophils and macrophages. Within these different leukocytes, intracellular fungal form varied, indicating that triggers in the intracellular milieu can override thermal morphological determinants. As in human talaromycosis, conidia were predominantly phagocytosed by macrophages rather than neutrophils. Macrophages provided an intracellular niche that supported yeast morphology. Despite their minor role in *T*. *marneffei* conidial phagocytosis, neutrophil numbers increased during infection from a protective CSF3-dependent granulopoietic response. By perturbing the relative abundance of neutrophils and macrophages during conidial inoculation, we demonstrate that the macrophage intracellular niche favours infection establishment by protecting conidia from a myeloperoxidase-dependent neutrophil fungicidal activity. These studies provide a new *in vivo* model of talaromycosis with several advantages over previous models. Our findings demonstrate that limiting *T*. *marneffei’s* opportunity for macrophage parasitism and thereby enhancing this pathogen’s exposure to effective neutrophil fungicidal mechanisms may represent a novel host-directed therapeutic opportunity.

## Author summary

For people with compromised immune systems, such as those suffering from AIDS, fungal infections are difficult to treat and often deadly. Different fungal species have different ways of avoiding destruction by the immune system during infection. For *Talaromyces marneffei*, the ability to infect host macrophages and replicate within them as yeast is thought to be key to their survival and spread throughout the human body. Here, we use a larval zebrafish infection model to study interactions between *T*. *marneffei* and cells of the innate immune system in greater detail than previously possible. We show that during early infection, *T*. *marneffei* spores are taken up primarily by macrophages. Limiting access to this macrophage niche enhanced engulfment and destruction of spores by neutrophils, another innate immune cell type important in host defences. This neutrophil antifungal activity was reduced in animals lacking Myeloperoxidase, an abundant antimicrobial enzyme of neutrophil granules, proving that myeloperoxidase is crucial for host defence against *T*. *marneffei*. These studies suggest that blocking access of infective *T*. *marneffei* spores to their macrophage niche, thereby exposing them to neutrophil fungicidal activity, may be therapeutically effective in *T*. *marneffei* infection.

## Introduction

Pathogenic fungal infections represent an important but widely overlooked human disease burden [1]. Invasive fungal infections, primarily affecting immunocompromised individuals, carry a high rate of mortality, despite the availability of antifungal drugs [2]. A uniting biological feature of a number of pathogenic fungi is dimorphism: modulation of morphological form in response to environmental cues. Most dimorphic fungal pathogens, such as *Blastomyces dermatitidis*, *Histoplasma capsulatum*, *Paracoccidioides brasiliensis* and *Talaromyces marneffei* (formerly *Penicillium marneffei*), exist in the environment in hyphal form, but convert to yeast growth during human infection [3–6]. Conversely, some dimorphic fungal pathogens, such as *Candida albicans*, exist as commensal yeasts, but under permissive conditions can cause invasive infection upon extension of germ tubes and subsequent hyphal growth [7]. Other common opportunistic fungal pathogens including *Cryptococcus neoformans*, which causes disseminated intracellular yeast infection leading to meningoencephalitis [8], and *Aspergillus fumigatus*, which causes serious pulmonary infections in immunocompromised patients [9] maintain a single morphological state despite having the capacity to change under certain circumstances, such as mating or development [10, 11].

Professional phagocytes of the innate immune system (neutrophils and macrophages) provide the first line of defence against fungal infection [12]. For *T*. *marneffei*, initial interactions are characterised by phagocytosis of conidia by leukocytes in the lung, followed by leukocyte-facilitated hematogenous dissemination [13]. For *Cryptococcus*, which infects as a yeast, macrophages also may play a role in pathogen dissemination [14]. Many fungal pathogens, such as *H*. *capsulatum* and *T*. *marneffei*, proliferate within macrophages as yeast [15–17], while invasive hyphae formed by *A*. *fumigatus* cannot be phagocytosed and elicit a neutrophil-dominated response, including generation of reactive oxygen species and formation of neutrophil extracellular traps (NETs)[18]. Although much has been learnt from mammalian infection models regarding disease progression, these models are inherently limited with regards to observing host-pathogen interactions *in vivo* and in real-time.

Zebrafish embryos and larvae provide an excellent platform for high-content imaging of early host-pathogen interactions, especially since the generation of transgenic strains labelling neutrophils [19–21] and macrophages [22–24]. The zebrafish toolbox has proven useful for modelling bacterial infection [25], particularly tuberculosis [26, 27]. Zebrafish have been utilised for modelling and imaging infections with the human fungal pathogens *Candida spp*. [28–31], *Aspergillus spp*. [32–35], *Cryptococcus spp*. [36–38], and *Mucor spp*. [39]. Such studies have provided significant new insights into the molecular and cell biology of host-pathogen interaction during these infections.

Here we present a new *in vivo* zebrafish model of talaromycosis (formerly called penicilliosis) that is caused by *T*. *marneffei*, a thermally dimorphic, opportunistic pathogen of humans. *T*. *marneffei* is capable of switching between a saprophytic hyphal growth form and a pathogenic yeast form in response to temperature and host cues including factors such as pH, salt concentration, calcium signalling and iron availability [40–43]. In the host, it primarily occupies the phagocyte niche but extracellular fungal cells are also evident, presumably due to host cell death.

For these studies, we have exploited the ectothermic nature of the zebrafish host. Although zebrafish are customarily held at 28°C in the laboratory, their normal development is documented for temperatures up to 33°C [44], and in the wild populations are found at temperatures up to 38.6°C [45]. We model an invasive hyphal form of *T*. *marneffei* infection unique to this zebrafish model (at 28°C) and a disseminated intracellular yeast form of infection resembling the human disease (at 33°C). These studies show that thermal dimorphism can be overridden by intracellular cues. We demonstrate a protective, G-CSFR-dependent expansion of neutrophils during protracted infection. Our studies particularly focused on the initial period of infection establishment, when fungal spores first encounter host leukocytes. These studies show that macrophages provide a protective niche for fungal conidia during infection establishment, whereas neutrophils exhibit a strongly fungicidal activity towards conidia that is myeloperoxidase dependent.

## Results

### Intravascular delivery of *T*. *marneffei* establishes a reproducible systemic invasive filamentous form of talaromycosis in zebrafish larvae at 28°C

**Fig 1:**
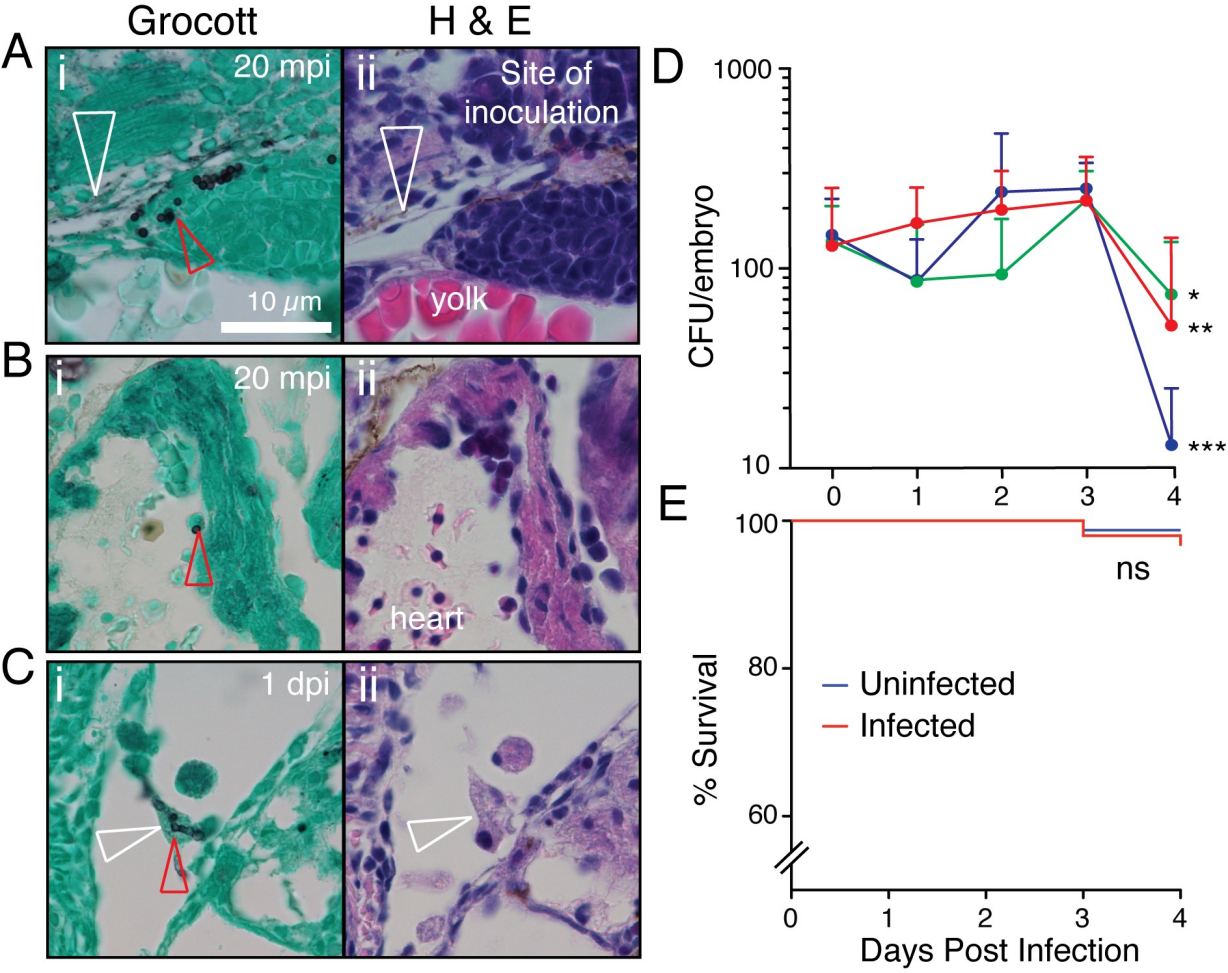
*T*. *marneffei* infection of zebrafish at 28°C. (A-C) Histological time-course following *T*. *marneffei* inoculation of zebrafish, stained by Grocott methanamine silver and Evan’s Blue counterstain (i), with adjacent hematoxylin and eosin-stained sections (ii). mpi, minutes post infection; dpi, days post infection. Red arrowheads indicate fungal conidia. White arrowheads indicate Duct of Cuvier (A) and leukocyte with intracellular fungal elements (C). (D) *T*. *marneffei* CFU time-course at 28°C following intravascular inoculation of target dose of approximately 150 fungal conidia (actual dose verified by 0 dpi CFU). Different colors indicate 3 independent experiments, mean±SEM, n≥5 embryos/group/experiment. *p = 0.016, **p = 0.0059, ***p = 0.0003 for statistical comparison between 3 and 4 dpi. (E) Embryo survival following intravascular inoculation in (D). Data are pooled embryos from 3 experiments: n = 474 uninfected, 339 infected. NS: not significant.

To optimise modelling of talaromycosis in zebrafish larvae, a variety of pathogen inoculation approaches and larval culture conditions were tested. At 28°C, immersion of 24 hpf embryos in *T*. *marneffei* conidia delivered within the chorion did not result in infection (0% (0/55) infected and 2% (1/56) death after 24 hours of co-incubation). However, reproducible local infection was established by intramuscular or 4^th^ ventricle injections, while systemic infection was initiated by intravenous inoculation into the Duct of Cuvier at 52 hpf. Histology demonstrated fungal conidia in close proximity to vascular walls immediately following inoculation (Fig 1A), their immediate phagocytosis by leukocytes (Fig 1B) and their germination within 1 day post infection (dpi), including within intravascular leukocytes (Fig 1C). From these initial dose-finding studies, it was established that intravenous inoculations of 100–150 viable conidia established a systemic infection that resulted in <25% mortality during the course of the experiment, with stable fungal colony-forming unit (CFU) counts until 4 dpi, when CFU counts declined (Fig 1D and 1E). This indicates that for infective challenges in this dose range, the zebrafish innate immune system alone is capable of controlling a *T*. *marneffei* infection. As expected, considering the thermal dimorphism of *T*. *marneffei* [3], during the later days of infection extension of fungal germ tubes within phagosomes elongated some leukocytes (Fig 1C and S1D Fig). In some embryos, there was also invasive filamentous growth within various tissues (S1C and S1H Fig), including the brain (S1E Fig). By 3–4 dpi, accumulation of leukocytes at infection foci resulted in abscess and/or a tight collection of cells reminiscent of the “early granuloma” observed in larval *M*. *marinum* infection [46] (S1C and S1F–S1H Fig). Although this form of invasive filamentous infection digressed from the predominantly yeast-form infection observed in human talaromycosis, it did confirm that *in vivo* infection *per se* did not provide sufficient cues for *T*. *marneffei* to switch to its yeast form.

### The intracellular environment of leukocytes can override thermal *T*. *marneffei* dimorphism signals at 33°C

**Fig 2:**
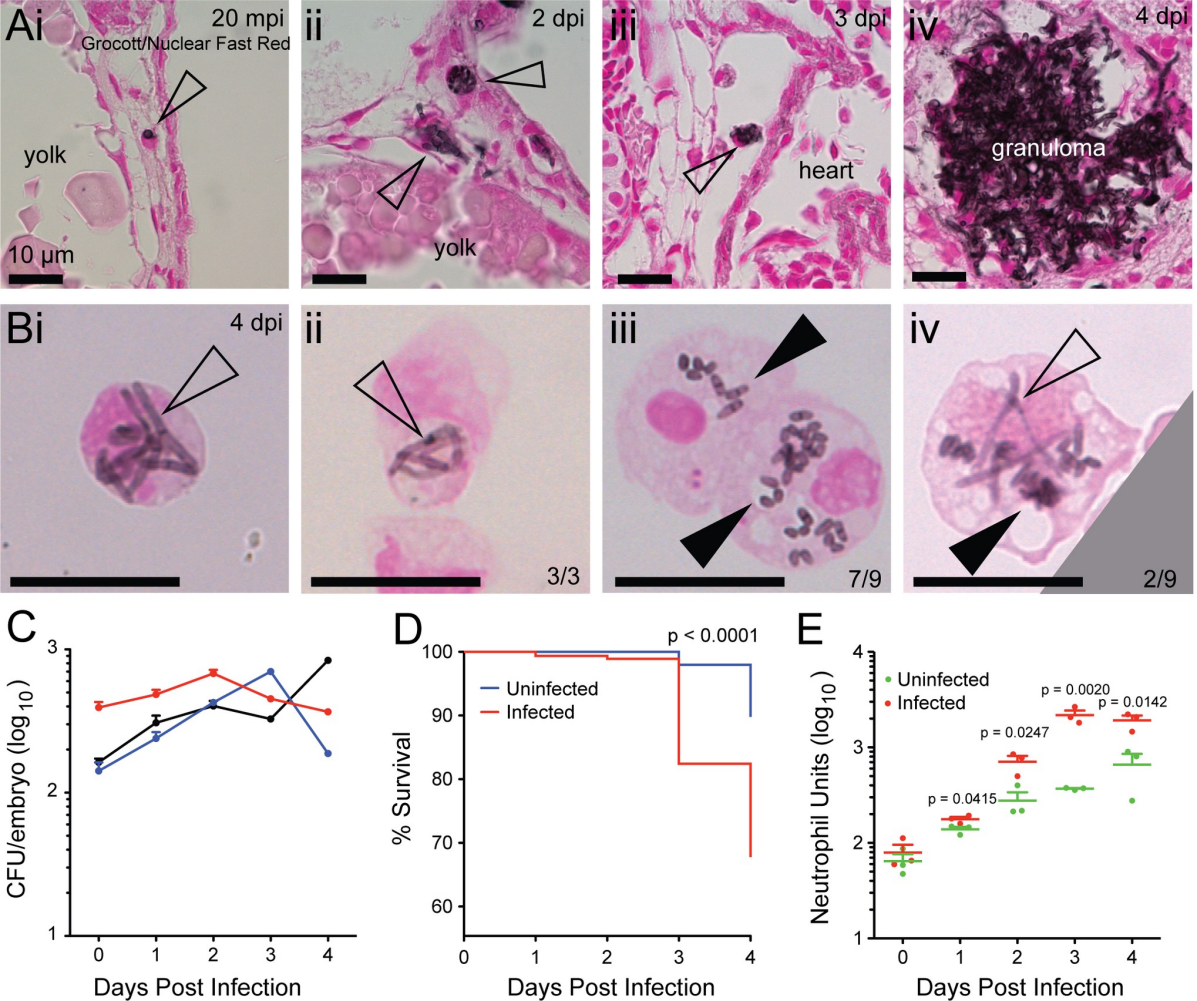
*T*. *marneffei* infection of zebrafish at 33°C. (Ai-iv) Histology of infected zebrafish embryos at different stages of *T*. *marneffei* infection. Sections were stained with Grocott methanamine silver to visualize fungi and counterstained with Nuclear Fast Red. Scale bar: 10 μm. (i) Phagocytosed conidium (arrowhead) within host leukocyte in the circulation at 20 minutes post infection. (ii) At 2 dpi, two fungal morphologies were observed: intracellular fungi assumed typical yeast morphology (upper arrowhead), while extracellular fungi displayed an elongated form (lower arrowhead). (iii) Yeast morphology of *T*. *marneffei* within a circulating infected leukocyte (arrowhead) at 3 dpi. (iv) Granuloma formation at 4 dpi. (B)FACS-purified, cytospun, *T*. *marneffei*-laden leukocytes prepared from infected embryos at 4 dpi, incubated at 33°C during infection. Grocott methanamine silver with Nuclear Fast Red counterstain. (i and ii) show neutrophils laden with hyphal forms (empty arrowheads), representative of all neutrophils observed. (iii) shows two macrophages laden with yeast forms (full arrowheads), the dominant morphology observed. (iv) shows a macrophage containing fungal cells of both yeast and hyphal morphologies. Scale bar: 10 μm. (C) Three representative CFU time-courses of infection at 33°C. In contrast to infections at 28°C (Fig 1D), no consistent decrease in CFU was observed at 4 dpi. Data are mean±SD of 3 independent replicates, n = 5 embryos (pooled)/timepoint/experiment. (D) Kaplan-Meier life table analysis for the treatment groups shown in (E). Significantly reduced survival occurred in infected groups compared to uninfected groups. n = 297 for uninfected, 319 for infected. P-value from Gerhan-Breslow-Wilcoxon Test. (E) Measurement of neutrophil populations during infection using Neutrophil Units. A significant increase in neutrophil population size occurred in the infected group compared to uninfected group over 1–4 dpi. Data are mean+SEM from 3 independent experiments. n = 5 embryos/group/timepoint in each experiment. P-values from unpaired two-tailed t-test.

To model infection with yeast morphology *T*. *marneffei*, as observed in human disease [47], infection was also established at 33°C [3] (Fig 2). This is a temperature which zebrafish tolerate well [26, 29, 44, 48–51], and at which the yeast morphological switch occurs *in vitro* [40]. Furthermore, it reflects the temperatures of human torso and extremity skin (31–34°C) [52–54], a tissue characteristically involved in disseminated talaromycosis and where intracellular yeast forms are readily found on biopsy [55, 56].

Despite an extensive literature of zebrafish experimentation at 33°C, we specifically verified experimentally that zebrafish phagocyte function is intact at 33°C. We conducted comparative experiments at 28°C and 33°C with *Aspergillus fumigatus*, a fungal pathogen that is not thermally dimorphic, and has been previously studied in zebrafish at 28°C [32–35]. We compared four parameters of phagocyte function at the two temperatures: (1) the initial migratory response of neutrophils and macrophages to the site of conidial inoculation (S2A Fig); (2) the initial phagocytic response of neutrophils and macrophages to *A*. *fumigatus* conidia following their arrival at the site of conidial inoculation (S2B Fig); (3) the myelopoietic response to inoculation with *A*. *fumigatus* conidia over a 4-day period (S3 Fig); (4) the impact of morpholino-induced perturbations of leukocyte abundance on the germination of *A*. *fumigatus* 24 hpi (S4 Fig). Furthermore, for scenarios (1–3), we conducted these experiments with both live conidia and dead conidia (killed by γ-irradiation) to address the possibility that, had any variation been observed between temperatures, that this might be due to different rates of conidial germination and/or proliferation at the two temperatures. For each of these 13 experimental scenarios, no consistent significant difference was observed in any endpoint (S2–S4 Figs). 7/53 pairwise comparisons testing the null hypothesis “that there was no temperature-dependent difference in the endpoint” generated a p-value (corrected for multiple comparisons) that was <0.05. We therefore concluded that phagocyte numbers and function were intact at 33°C and that experiments conducted at 33°C would make a valid contribution to an exploration of the role of phagocyte function in the pathogenesis of *T*. *marneffei* infection establishment.

For infections that proceeded at 33°C, histology again demonstrated leukocyte conidia phagocytosis (Fig 2Ai) and *T*. *marneffei* growth within leukocytes and tissues (Fig 2Aii–2Aiv). Cytospun leukocyte preparations at 4 dpi of 33°C infections demonstrated germinated, proliferating fungal cells within leukocytes. Strikingly, at this temperature, *T*. *marneffei* assumed an elongated, septate filamentous form within all observed neutrophils, whereas within macrophages, fission yeast morphology (recognized as characteristic elongated forms with a medial septum [57]) predominated (7/9 macrophages) (Fig 2B). We confirmed that also in mammalian macrophages at 33°C, *T*. *marneffei* assumed this characteristic fission yeast morphology (S5 Fig). Collectively, these morphologically-based observations indicate that the different intracellular milieu of each phagocyte type can differentially influence *T*. *marneffei* form, and that at 33°C, the macrophage milieu favours the transition to the yeast form.

At 33°C, despite a vigorous granulopoietic response to infection (Fig 2E), fungal CFU numbers did not decrease at 4 dpi (Fig 2C), and death from infection was increased (Fig 2D), suggesting enhanced fungal viability at this temperature.

### Sustained talaromycosis triggers a protective myelopoietic response

**Fig 3:**
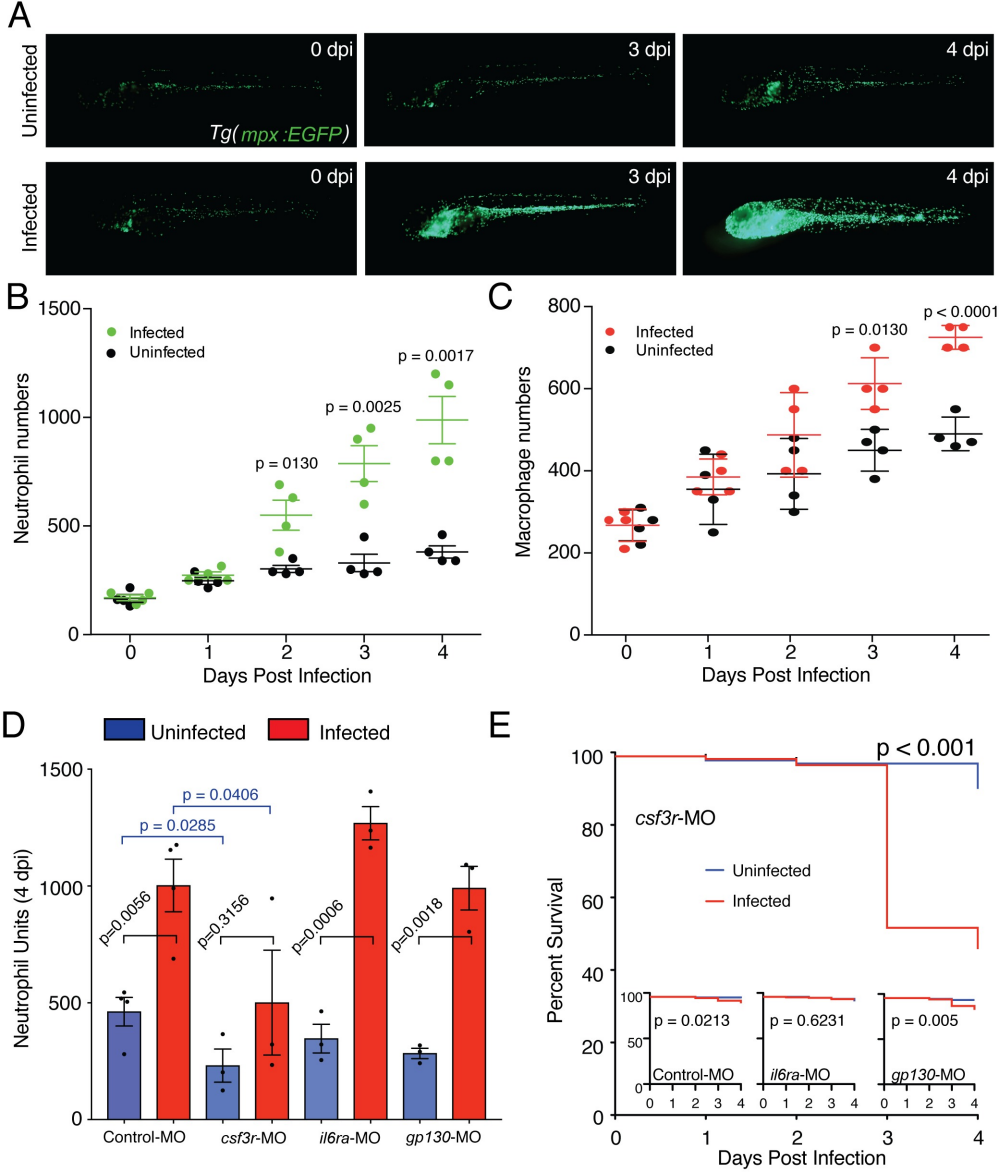
Zebrafish mount a Csf3r-dependent granulopoietic response to *T*. *marneffei* infection. (A) Low magnification images of infected and uninfected whole *Tg(mpx*:*EGFP)* embryos at 0, 3 and 4 dpi, displaying the profound infection-driven increase in EGFP-expressing neutrophil numbers. (B, C) Neutrophil (B) and macrophage (C) population expansion in infected versus uninfected embryos from 0–4 dpi. These data derive from enumerating neutrophil and macrophage numbers in the same embryos, of genotype *Tg(mpx*:*EGFP/mpeg1*:*Gal4FF/UAS-E1b*:*Eco*.*nfsB-mCherry)*. Data displayed are mean±SD, n = 4 embryos/group. These data replicate other data derived from separately scoring these leukocyte populations in different embryos, in n≥2 independent other experiments. (D) Neutrophil population sizes at 4 dpi represented by Neutrophil Units in embryos injected with antisense morpholino oligonucleotides targeted to knockdown *csf3*-receptor (*csf3r*-MO) or interleukin-6 receptor (*il6ra*-MO and *gp130*-MO) Data are mean±SEM, n = 10 embryos/group/experiment, n≥3 experiments. Intragroup +/- infection p-values from 2-tailed unpaired t-test; p-values from intergroup comparisons involving *csf3r*-MO from 1-tailed unpaired t-test, given the *a priori* expectation of a reduction in neutrophil values in *csf3r*-deficiency [65, 95] (E) Kaplan-Meier life table analysis for the treatment groups shown in (C). Numbers/group (uninfected/infected): Control MO, 474/326; *csf3r*-MO, 443/233; *il6ra*-MO, 462/330; *gp130*-MO, 465/313. P-values from Gerhan-Breslow-Wilcoxon Test.

To facilitate live imaging of host-pathogen interactions, infection was performed using larvae expressing neutrophil- and/or macrophage-specific fluorescent transgenes. Quantification of leukocyte numbers over 4 days of infection at 28°C revealed a dramatic increase from 2–4 dpi in both neutrophil and macrophage numbers in response to active infection (Fig 3A–3C). By 4 dpi, macrophage and neutrophil numbers had increased to levels 150% and 260% respectively of those in uninfected controls, while pathogen numbers decreased by 65% over the same period (Fig 1D), indicating that the vigorous myelopoietic response was a component of an effective host response that controlled the infective burden. A vigorous granulopoietic response was also seen over 4 days of infection at 33°C (Fig 2E).

### The protective granulopoietic response is CSF3R (G-CSFR)-dependent

Amplification of leukocyte numbers during zebrafish bacterial infection has been shown to depend on signalling through the Csf3-Csf3r pathway [58], while both Csf3 and Interleukin-6 are known to be important in the response to yeast infection in mammalian systems [59, 60]. To interrogate these pathways in the context of talaromycosis, Csf3 signaling was intercepted by knocking down the single chain of the homodimeric *csf3* receptor (*csf3r*), and interleukin-6 signalling was intercepted by knockdown of each subunit of the heterodimeric interleukin 6 receptor (*il6ra* and *gp130*). To quantify granulopoiesis, infections were performed in *Tg(mpx*:*EGFP)* larvae at 28°C and quantified as previously described [61].

*Csf3r* knockdown significantly reduced baseline neutrophil numbers and also significantly reduced the increase in neutrophil population size at 4 dpi (Fig 3D). The relative increase in neutrophil abundance in both control and *csf3r-*knockdown infected embryos was 2.2-fold. Hence the significant difference in the absolute increase is likely to have in part reflected the significantly lower basal neutrophil abundance in *csf3r*-knockdown embryos. This difference was not due to a different pathogen burden following infection establishment in the face of lower basal neutrophil abundance, since *csf3r* knockdown did not alter fungal survival/proliferation as reflected by 24 hours post infection (hpi) CFU numbers (Fig 5B). By 4 dpi, the significantly reduced granulopoietic response of infected *csfr3*-knockdown embryos resulted in impaired survival from infection (Fig 3E).

In contrast, *il6r* subunit knockdown did not impair the infection-driven granulopoietic response (Fig 3D). The effect of *il6r* subunit knockdown on basal neutrophil population size was also modest.

Collectively, these data indicate that the large expansion of neutrophil numbers during sustained *T*. *marneffei* infection is dependent on an interaction between pathogen and host, which mounts a cytokine-driven granulopoietic response that is in part *csf3r*- but not *il6r*-dependent. Intact basal *csfr3* signalling is required for effective, protective, host-defences to talaromycosis.

### Phagocytosis of conidia during infection establishment is dominated by macrophages

**Fig 4:**
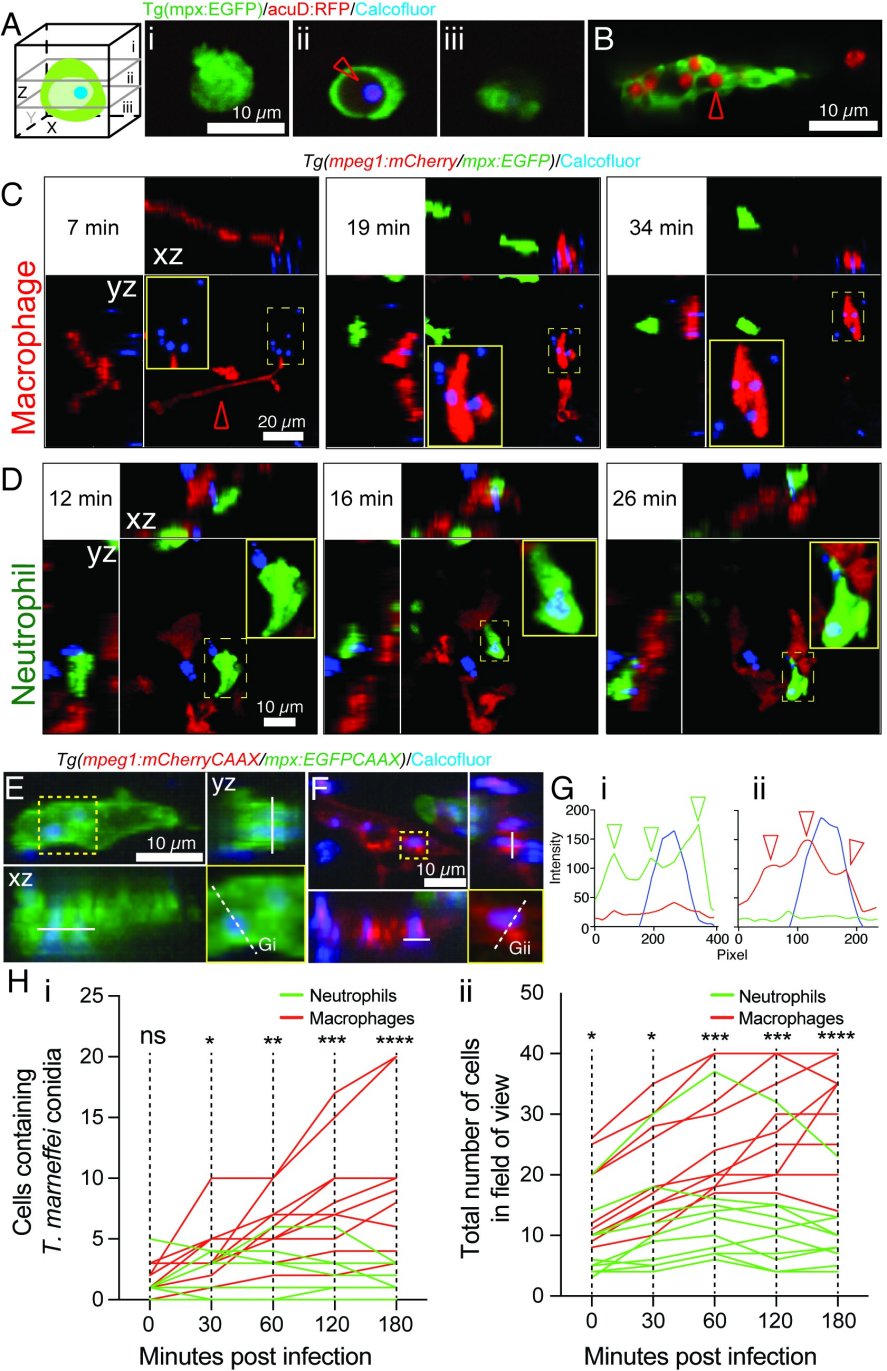
Macrophages dominate *T*. *marneffei* phagocytosis during infection establishment. (A, B) Confocal imaging of leukocytes and calcofluor-stained acuD:RFP fungal conidia in *Tg(mpx*:*EGFP)* fluorescent leukocytes. (A) shows a germinated, RFP-expressing, blue calcofluor-stained conidium (red arrow) within a non-fluorescent vacuole of a bright EGFP^high^ neutrophil. Z-stack sections (i-iii) orientated as in schematic diagram. (B) shows a dull EGFP^low^ macrophage laden with multiple RFP-expressing conidia. (C,D) Phagocytosis of calcofluor-stained fungal conidia by a macrophage (C) and neutrophil (D) in *Tg(mpeg1*:*mCherry/mpx*:*EGFP)* embryo (C) inoculated in a somite. The still images are from S1 and S2 Movies respectively. The macrophage extends a long process towards the conidium (red arrowhead); conidial phagocytosis is followed by macrophage movement over the site of phagocytosis and out of the field. In each panel, maximum projection z-stack image is supplemented by xz and yz projections, orientated as in the schematic diagram. (E-G) Confocal imaging of intracellular calcofluor-stained conidia within a neutrophil (E) and macrophage (F) of a *Tg(mpeg1*:*mCherryCAAX/mpx*:*EGFPCAAX)* embryo, with dimensional projections as in (C). In each case, the lower right yellow-boxed panel is a detail of the yellow-boxed area in the upper left xy maximum intensity projection, displaying the cross-section (white dashed line) for which a fluorescence intensity channel profile is shown in G. Arrowheads indicate peaks of fluorescence corresponding to neutrophil (Gi, green) and macrophage (Gii, red) cellular membranes. (H) Enumeration of conidia phagocytosis (i) by macrophages (red lines) and neutrophils (green lines) reflects higher macrophage recruitment (ii) following somite infection. Lines represent individual infected zebrafish followed over time. N = 10 embryos/group imaged on n = 8 different days. P-values from Mann-Whitney test: ns = not significant, * = <0.05, ** = <0.01, *** = <0.001, **** = <0.0001.

Since leukocyte/conidia interactions were a prominent histological feature of the initial host response to *T*. *marneffei* conidia inoculation, they were examined in detail at 28°C (Fig 4). For these experiments, we used intramuscular inoculations, as we were not testing hypotheses about the normal route of infection, but rather about the direct interaction between conidia and leukocytes. These studies were conducted at 28°C, as the conidial state of *T*. *marneffei* at the time of inoculation is not temperature dependent, and this is the temperature at which zebrafish leukocyte function is usually characterised.

We confirmed that leukocytes actually phagocytosed fungal conidia, rather than merely associated with them. Fig 4A shows a neutrophil with a calcofluor-stained, germinated conidium, proven by z-stack analysis to be within an intracellular vacuole. Fig 4B shows a GFP-low macrophage in the *Tg(mpx*:*EGFP)* line (as previously described [62]) containing multiple germinated conidia. Using reporter lines in which red and green fluorophore expression is driven in macrophages and neutrophils respectively, active interaction of both neutrophils and macrophages with calcofluor-stained conidia was documented in the initial stages of infection (Fig 4C and 4D and S1–S3 Movies), with apparent phagocytosis. Using new reporter lines in which reporter fluorophore expression was targeted to membranes by linkage to the CAAX prenylation signal, Z-stack profiling confirmed that conidia associated with leukocytes were intracellular and within membrane-lined phagosomes, as demonstrated by cross-sectional fluorescence intensity profiles (Fig 4E–4G).

Although both neutrophils and macrophages were capable of phagocytosing *T*. *marneffei* conidia, following both somitic (Fig 4G) and intravascular inoculation (S6 Fig), *T*. *marneffei* conidia were phagocytosed almost exclusively by macrophages (Fig 4H). To quantify the various leukocyte-pathogen interactions at the inoculation site, fluorescence signal colocalization were analyzed based on the different leukocyte-associated reporter fluorophores and blue fluorescence of calcofluor-labelled conidia. Analysis of the caudal hematopoietic tissue of *Tg(mpx*:*EGFP/mpeg1*:*mCherry*) embryos 2 hours following intravenous calcofluor-labelled conidia inoculation showed that, while the neutrophil:macrophage voxel ratio was 1:2.6 (S6A Fig), reflecting the relative population sizes of the two phagocyte types, the ratio of conidia associated with these neutrophils and macrophages was 1:60 (S6B Fig).

In contrast, following intramuscular inoculation, although *T*. *marneffei* conidia were still preferentially phagocytosed by macrophages, neutrophils also actively engaged in conidial phagocytosis (Fig 4Hi). Hence, although both neutrophils and macrophages phagocytose *T*. *marneffei* conidia during the initial stages of infection, macrophage phagocytosis predominates.

### During infection establishment, neutrophils are fungicidal, while macrophages provide a protective niche

Despite a common function as professional phagocytes, neutrophils and macrophages utilize distinctive antimicrobial arsenals [63], and hence display diverse antimicrobial properties that are often microbe-specific. Given the specific effects of the neutrophil and macrophage intracellular milieu on *T*. *marneffei* form at later stages of infection (Fig 2B), we hypothesized that neutrophils and macrophages might also play divergent roles during infection establishment.

**Fig 5:**
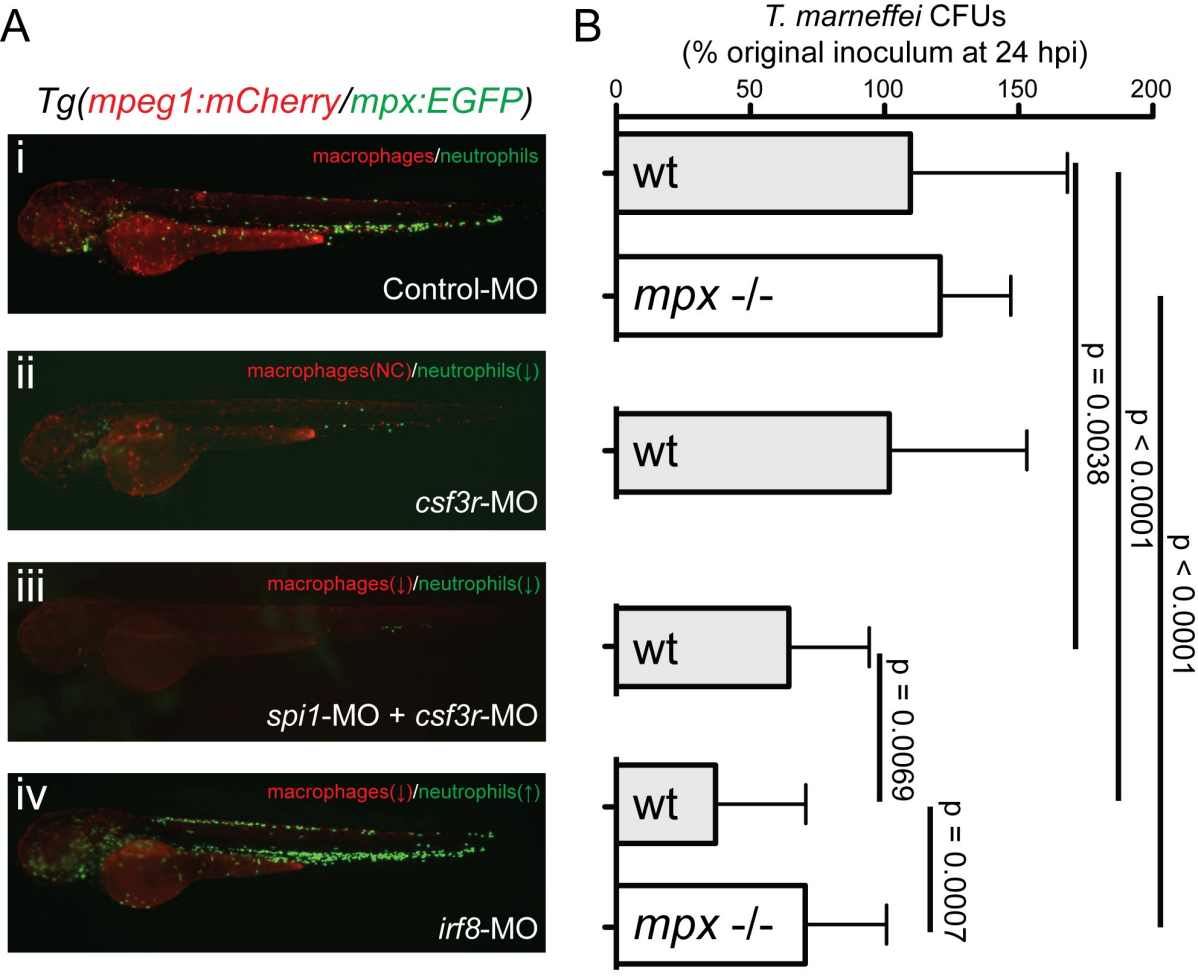
Neutrophils and macrophages play opposing roles during establishment of *T*. *marneffei* infection. (A) Representative images of 52 hpf *Tg(mpeg1*:*mCherry/mpx*:*EGFP)* embryos injected with antisense morpholino oligonucleotides to perturb the balance of neutrophil and macrophage populations. (B) *T*. *marneffei* CFU numbers at 24 hpi corresponding to the aligned treatment groups in panels (Ai-iv), for wild-type (WT) and myeloperoxidase-deficient (*mpx*^-/-^) genotypes as shown. Data are mean±SEM for n≥3 experiments, n≥5 embryos/group/experiment.

To determine the different roles of neutrophils and macrophages during the initial stages of *T*. *marneffei* infection, CFU counts were compared at 0 and 24 hpi in embryos at 28°C that had been experimentally manipulated to modulate the relative numbers of neutrophils and macrophages (Fig 5).

Viable fungal cell number did not change between 0 and 24 hpi in leukocyte-replete control embryos (109.7% of baseline on average at 24 hpi) (Fig 5Ai and 5B), indicating either that conidia are both non-proliferative and resistant to killing, or that fungal death and proliferation were balanced in this context.

In embryos depleted of both neutrophils and macrophages by *spi1+csf3r*-knockdown using antisense morpholino oligonucleotides [64], 24 hpi *T*. *marneffei* CFU counts were significantly reduced to 64.4±6.7% of baseline (Fig 5Aiii and 5B). This demonstrated both that the presence of leukocytes during infection establishment enhanced conidial survival and/or fungal proliferation, and indicated the existence of a leukocyte-independent fungicidal activity.

In *csf3r* morphant embryos, which are depleted of neutrophils with little effect on macrophages [65, 66], 24 hpi CFU counts were 101.8±10.3% of baseline (Fig 5Aii and 5B), indicating that the presence of normal numbers of macrophages alone is sufficient to protect *T*. *marneffei* conidia from the leukocyte-independent fungicidal activity observed in *spi1+csf3r* morphants.

To complement the phagocyte depletion experiments, the effects of expansion of neutrophil populations on *T*. *marneffei* viability was examined in scenarios of different macrophage abundance. As previously reported [67], overexpression of *csf3b* from mRNA injection relatively selectively increased neutrophil numbers. In our hands, it resulted in approximately twice as many neutrophils at 2 dpf (S7Ai and S7Aii Fig) compared to controls, but only an increase of 39% in macrophages (S7Aiii and S7Aiv Fig) [68]. No significant change in fungal viability was observed over the first 24 hpi for embryos overexpressing *csf3b* (S7B Fig), indicating that the slightly increased macrophage population present in these embryos is sufficient to provide for conidial viability, even when neutrophil populations are expanded.

Knockdown of *irf8* expands neutrophil numbers, but at the expense of macrophage numbers [61, 69]. In *irf8* morphants, CFU counts at 24 hpi were 37.1±6.7% of baseline, which was significantly lower than for both control animals and *spi1+csf3r* morphants (Fig 5B), pointing to a potent fungicidal activity of the expanded neutrophil population when availability of the protective macrophage niche is reduced.

Collectively, all four scenarios support the hypothesis that macrophages provide a protective niche for *T*. *marneffei* conidia during infection establishment, shielding conidia from both neutrophil-dependent and leukocyte-independent fungicidal mechanisms.

In agreement with the important role played by neutrophils in controlling infection at later timepoints (Fig 3E), neutrophils were found to be strikingly fungicidal. As neutrophils were clearly the more fungicidal leukocyte during establishment of *T*. *marneffei* infection, we examined potential mechanisms that might determine neutrophil response to this pathogen and its outcome.

### Neutrophil fungicidal activity requires myeloperoxidase

Myeloperoxidase is an abundant neutrophil enzyme important for generating potent antimicrobial radicals, and is critical for defence against other fungal pathogens in mammals [70–72]. We therefore hypothesized that neutrophil-dependent fungicidal activity against *T*. *marneffei* was dependent on myeloperoxidase activity. We tested the requirement for myeloperoxidase (*mpx*) using an *mpx*-deficient zebrafish mutant that is neutrophil-replete but lacks enzymatic Mpx activity [73].

During the establishment phase of infection, there was no difference in *T*. *marneffei* CFU counts at 24 hpi between WT and *mpx*^-/-^ larvae for infections at both 28°C and 33°C (Fig 5B and S8A Fig) despite equivalent neutrophil populations over this period (S8B Fig). However, the enhanced fungicidal activity of the expanded neutrophil population in *irf8*-MO embryos, which reduced CFU counts at 24 hpi, was lost in *mpx*^-/-^ embryos (Fig 5B).

During sustained infection, *mpx*^-/-^ embryos mounted a vigorous granulopoietic response which is >2.5-fold higher than that of WT embryos at 3 dpi (S8B Fig). However, despite this expanded neutrophil population, *mpx*^-/-^ embryos carried a fungal CFU burden similar to WT embryos (S8A Fig).

Collectively, these data indicate that the fungicidal activity of neutrophils against *T*. *marneffei* is in part myeloperoxidase-dependent, and that this component of neutrophil antifungal activity becomes most critical when the neutrophil population is expanded and macrophage abundance is depleted.

### Depleting the macrophage niche enhances clearance of conidia during infection establishment

During infection establishment, our results support a model in which *T*. *marneffei* preferentially parasitizes macrophages, which provide an intracellular niche that shields conidia from both neutrophil-dependent and neutrophil-independent anti-fungal mechanisms. We therefore hypothesized that limiting access to the macrophage niche would enhance conidial clearance during infection establishment.

**Fig 6:**
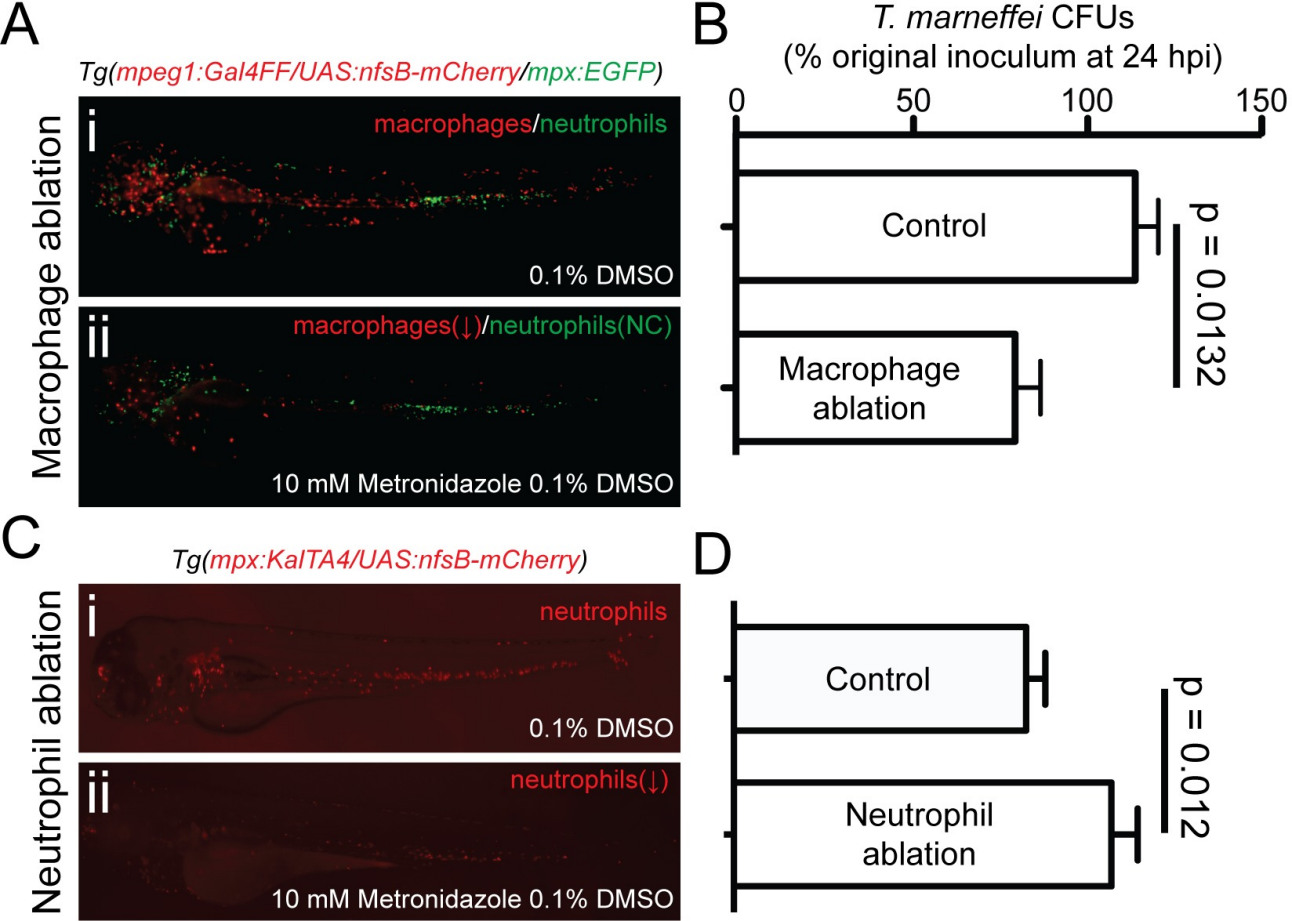
Depletion of the macrophage niche increases conidial destruction during establishment of infection. (A) Ablation of macrophages decreases conidial viability in the first 24 hpi. (i) and (ii) show representative images of nitroreductase-dependent metronidazole-mediated macrophage ablation following treatment of transgenic embryos (ii) versus untreated control embryos (i). Ablation efficiency is quantified in (S9A Fig). (B) *T*. *marneffei* CFU numbers at 24 hpi in macrophage-replete control (*Tg(mpeg1*:*Gal4FF/UAS-E1b*:*Eco*.*nfsB-mCherry)* negative, treated with 10 mM metronidazole) compared to macrophage-depleted (*Tg(mpeg1*:*Gal4FF/ UAS- E1b*:*Eco*.*nfsB-mCherry)* positive, treated with 10 mM metronidazole). (C) Ablation of neutrophils increases conidial viability in the first 24 hpi. (i) and (ii) show representative images of Nitroreductase-dependent metronidazole-mediated neutrophil ablation in treated (ii) versus diluent-treated control embryos (i). Ablation efficiency is quantified in (S9B and S9C Fig). (D) *T*. *marneffei* CFU numbers at 24 hpi in neutrophil-replete control (*Tg(mpx*:*KalTA4/UAS-E1b*:*Eco*.*nfsB-mCherry)* negative, treated with 10 mM metronidazole) compared to neutrophil-depleted (*Tg(mpx*:*KalTA4/ UAS- E1b*:*Eco*.*nfsB-mCherry)* positive, treated with 10 mM metronidazole) Data are mean±SEM, n≥5 embryos/group/experiment, n≥3 experiments.

To test this hypothesis, macrophages were ablated prior to infection at 28°C using the metronidazole-dependent nitroreductase system [74, 75]. Metronidazole treatment of *Tg(mpeg1*:*Gal4FF/UAS-E1b*:*Eco*.*nfsB-mCherry)* embryos reduced macrophage abundance by 70% (Fig 6A, S9A Fig and S4 Movie), which resulted in a significant (35%) reduction of *T*. *marneffei* CFU counts at 24 hpi, compared to control sibling embryos that were metronidazole-treated but did not carry the *UAS-E1b:Eco.nfsB-mCherry* transgene (Fig 6B).

Conversely, selective ablation of neutrophils by metronidazole treatment of *Tg(mpx*:*KalTA4/UAS-E1b*:*Eco*.*nfsB-mCherry)* embryos resulted in a 20% increase in fungal viability compared to controls (Fig 6C and 6D, S9B and S9C Fig), further supporting our findings that neutrophils exhibit fungicidal properties during infection establishment.

Taken together, these results suggest that limiting access to the macrophage intracellular niche may be one targetable pathway for restricting establishment of infection for the benefit of the host.

## Discussion

This new model of *Talaromyces marneffei* infection in larval zebrafish holds many advantages over current murine *in vivo* models [76, 77]. It combines a replete vertebrate innate immune system with transgenically labelled lineages and optical transparency to facilitate detailed live imaging of host-pathogen interactions. Additionally, the ectothermic biology of zebrafish allows experimental separation of thermal and host-dependent influences on fungal morphology. While this can also be achieved using the recently developed *Galleria mellonella* and *Caenorhabditis elegans* models [78, 79], the invertebrate hemocyte provides only limited insight into complex innate immune responses such as those described here.

Combining an ectothermal host and a thermally dimorphic pathogen in an experimental system modelling a human infection provides both opportunity and raises some technical questions. Although zebrafish experiments are normally conducted at 28–28.5°C, zebrafish physiology is robust at 33°C. Evidence for this is: existence of wild *D*. *rerio* populations in waters up to 38.6°C [45]; normal embryological development over this temperature range [44]; experimental studies involving human xenotransplantation [49]; conditional temperature sensitive mutant alleles [26, 50, 51]; and even temperature shifts employed in previous infection modelling studies [26, 29]. We provide new experimental evidence showing that the myeloproliferative and functional phagocyte response to *A*. *fumigatus*, a non-thermally dimorphic fungal pathogen, is similar at 28°C and 33°C.

*T*. *marneffei* exhibits thermal dimorphism, but even so, the fungal to yeast shift is not absolute at 37°C. At 37°C *in vitro*, the rate of hyphal conversion to yeast varies from 40% to 90% between the lowest and highest tolerated pHs, and NaCl concentrations ≥4% suppress hyphal to yeast conversion almost completely [40]. Even in human infection at 37°C, extracellular forms characteristically assume an elongated shape [47, 80]. As is uniquely possible in an ectothermic model, we exploited the ectothermic biology of the zebrafish host to identify a divergence between fungal morphology and temperature, specifically in response to the leukocyte intracellular milieu. The phase transition to a yeast morphology during human *T*. *marneffei* infection at 37°C is widely regarded to be triggered primarily by the ambient temperature, with the thermal dimorphism of the pathogen seen as an evolved pathogenic trait [42]. At 33°C, we observed filamentous forms in neutrophils and tissues (Fig 2Aii, 2Aiv, 2Bi and 2Bii), and predominantly yeast forms within macrophages (Fig 2Biii and 2Biv), providing strong evidence that the intracellular milieu of the phagocytosing leukocyte type may be a determinant of *T*. *marneffei* form *in vivo* that can override the influence of ambient temperature. We therefore hypothesize that one factor contributing to the macrophage being the preferred infectious niche for *T*. *marneffei* is its temperature-independent support of a phase transition to the more pathogenic yeast form.

In response to prolonged infection, we observed expansion of leukocyte populations, particularly neutrophils, and demonstrated that this protective response was at least in part dependent on signalling through G-CSFR. This demand-driven hematopoietic response to infection appears to be conserved, and has been since reported by others in response to *Salmonella enterica* infection [58]. Overexpression of the zebrafish Csf3r receptor ligand Csf3b by mRNA overexpression phenocopied the granulopoietic response observed during *T*. *marneffei* infection, further supporting our findings.

Genetic manipulation of leukocyte lineage numbers prior to infection demonstrated that macrophages provide a protective niche for fungal conidia, while neutrophils are fungicidal. Further, using an existing mutant, we demonstrated that the fungicidal activity of neutrophils was myeloperoxidase-dependent. Myeloperoxidase deficiency is a prevalent congenital human disorder of neutrophils with an incidence of 1:2000 [81]. Epidemiological studies associate myeloperoxidase-deficiency with increased risk of fungal infection [82], myeloperoxidase-deficient mice are vulnerable to *Candida* infection [83], and myeloperoxidase-deficient neutrophils display reduced ability to produce fungicidal neutrophil extracellular traps (NETs) [84]. Our demonstration of myeloperoxidase deficiency as a talaromycosis disease-enhancer in zebrafish suggests it may be an unrecognized disease-modifier of human talaromycosis and invites an evaluation of myeloperoxidase status in such patients.

Selective depletion of macrophages using the metronidazole system demonstrated that removal of this protective niche exposes *T*. *marneffei* conidia to neutrophil-dependent and independent antifungal mechanisms. Temporary depletion of macrophages in patients that transiently selectively reduce access of conidia to the macrophage niche might provide a novel therapeutic strategy to restrict infection establishment. We also hypothesize that transient macrophage ablation therapy in established talaromycosis may facilitate talaromyces infection eradication by exposing the organism to fungicidal neutrophils.

One limitation of this zebrafish model is that the infection is established by inoculation, rather than via alveolar macrophages which is presumed to be that natural route of infection [85]. However, in human *T*. *marneffei* infection, fungal forms are found in tissue macrophages throughout the body, including particularly in skin [56], lymph nodes [86] and bone marrow [87], and so our observations of the behaviour of tissue macrophages provide relevant insights to understanding the pathogenesis of the human disease.

Findings from this new *in vivo* zebrafish *T*. *marneffei* infection model have implications not only for talaromycosis but also for the pathogenesis of infections with other fungal and dimorphic pathogens including histoplasmosis, blastomycosis and coccidioidomycosis. Furthermore, it is a unique resource for exploring in detail the *in vivo* cell biology of leukocyte-pathogen interactions during this infection.

## Materials and methods

### Zebrafish

Zebrafish strains were: wildtype (AB*); *durif*(*mpx*^-/-^)^*gl8*^ [73]; *Tg(mpx*:*EGFP)*^*i113*^ [19]; *Tg(mpeg1*:*Gal4FF)*^*gl25*^ [22]; *Tg(mpeg1:mCherry)^gl23^* [22]; *Tg(mpx*:*Kal4TA4)*^*gl28*^ [88]; *Tg(UAS-E1b*:*Eco*.*nfsB-mCherry)*^*c264*^ (Zebrafish International Stock Centre, Eugene, OR). The new *Tg(mpeg1*:*mCherryCAAX)*^*gl26*^ and *Tg(mpx*:*EGFPCAAX)*^*gl27*^ lines were generated using Multisite Gateway cloning (Invitrogen) in combination with 1.87 kb of *mpeg1* promoter [22] and 8.35 kb of *mpx* promoter [20]. Fish were held in the Walter and Eliza Hall Institute and FishCore (Monash University) aquaria using standard practices. Because zebrafish exhibit juvenile hermaphroditism, gender balance in embryonic and larval experiments was not a consideration [89]. Embryos were held in egg water (0.06 g/L salt (Red Sea, Sydney, Australia)) or E3 medium (5 mM NaCl, 0.17 mM KCl, 0.33 mM CaCl_2_, 0.33 mM MgSO_4_, equilibrated to pH 7.0); from 12 hpf, 0.003% 1-phenyl-2-thiourea (Sigma-Aldrich) was added.

### Ethics and biosafety statement

Animal experiments followed appropriate NHMRC guidelines and were conducted under protocols approved by Ethics Committees of the Walter and Eliza Hall Institute (2007.004 and 2009.031) and Monash University (MAS/2010/18). In accordance with the approved protocol requirements, all zebrafish embryos and larvae used in experiments were younger than 7 dpf (i.e. experiments were concluded on the 6^th^ dpf which was the 4^th^ day after infection). We performed the experiments under Institution Biosafety Committee Notifiable Low Risk Dealing (NLRD) approvals 2007.01 (Walter and Eliza Hall Institute) and PC2-N23-10 (Monash University). *T*. *marneffei* was assigned to Risk Group 2 at the time these approvals were granted. In most jurisdictions, including endemic regions, *T*. *marneffei* is a risk group 2 organism.

### Talaromyces marneffei

*T*. *marneffei* strains, derived from the FRR2161 type strain, were: *acuD*:RFP strain, which expresses RFP on germination and the control strain SPM4 [90]. To prepare cells for injection, *T*. *marneffei* conidia were inoculated onto Sabouraud Dextrose (SD) medium and cultured at 25°C for 10–12 days when the cultures were conidiating. Conidia were washed from the plate with 0.005% Tween 80 solution, filtered, sedimented (6000 rpm, 10 min), resuspended in dH_2_O and stored at 4°C. For inoculation, conidia were resedimented and resuspended in PBS. Heat-inactivation and calcofluor staining was as described previously [22]. Calcofluor staining did not affect conidial viability, as evidenced by their subsequent germination *in vivo* (S1A Fig).

*T*. *marneffei* colony forming unit (CFUs) numbers per embryo were determined by thorough homogenization of individual embryos in 500 μL of dH_2_O using a Dounce homogenizer. 250 μL of homogenate was cultured on SD medium agar with 1% ampicillin for 3–4 days at 37°C. Plates were then incubated overnight at room temperature, and colonies that underwent yeast to hyphal morphological switching were scored as *T*. *marneffei* colonies.

### Zebrafish infection with *Talaromyces* *marneffei*

For inoculation, 52 hpf tricaine-anesthetized embryos were mounted on an agar mould with head/yolk within the well and tail laid flat on the agar. The *T*. *marneffei* conidial suspension was inoculated using a standard microinjection apparatus and large-bore needle via the common cardinal vein for systemic infection, or the 4^th^ ventricle or a somite aligned to the yolk extension tip for local infection [22, 91]. Inoculated embryos were held at 28°C or 33°C according experimental design. The delivered conidial dosage was determined by immediate CFU enumeration on a group of injected embryos. Following initial dose-finding experiments that established an intravascular inoculum of 100–150 CFU/embryo achieved <25% mortality for 28°C infections (Fig 1D and 1E), this was the target inoculum dose.

### Zebrafish infection with *Aspergillus fumigatus*

Freshly-prepared *A*. *fumigatus* conidia stocks (strains CEA10 and 295) for these experiments were stored at 4°C for < 2 months. Fresh aliquots were prepared for microinjection as described for *T*. *marneffei* spores, delivering a target inoculum of 50–150 live spores, verified by back-plating as described above for *T*. *marneffei*. To prepare dead *A*. *fumigatus* conidia for microinjection, they were γ-irradiated with 10kGy (delivered over 207 hr 27 min 10 sec) from the Monash University Gammacell 40 Exactor (Theratronics) with two Caesium-137 sources (dose selected based on [92]). Irradiated spores were verified as dead by plating and incubation for 5 days: no growth occurred. The dead spores were microinjected at the same dilution of stock as used for live spores. Irradiated spores still stained well with calcofluor. For intravascular delivery experiments, microinjection of conidia utilised polydimethylsiloxane (PDMS) microstructured surface arrays [93], while imaging was performed following mounting in PDMS imaging devices, as previously described [94].

### Leukocyte enumeration

Leukocyte numbers were determined by two techniques. For direct enumeration, manual counting of neutrophil numbers was assisted by the brush tool in Paintbrush 2.1.2 (Soggy Waffles), which records clicks to avoid duplicate counting. Alternatively, “Leukocyte Units” (LUs), a surrogate parameter proportional to leukocyte numbers determined by analysis of digital images, were computed as previously described and validated [61]. LUs incorporate an internally-controlled correction for cell size, and can be independently applied to the signal from fluorescent neutrophils and macrophages. Where appropriate, LUs are called “Neutrophil Units” or “Macrophage Units”. In some cases as indicated, leukocytes were enumerated in the tail region distal to the tip of the yolk extension, in order to score cells in a representative part of the whole animal where overlap and anatomical shape did not interfere with accurate scoring. Migrating phagocyte numbers and their phagocytosis of conidia were counted manually in reconstructed 3-dimensional imaged volumes using Imaris v5 (Bitplane).

### Gene knockdown by antisense morpholino oligonucleotides

Antisense morpholino oligonucleotides (MOs) were purchased from Gene Tools, LLC (Eugene, OR) (S1 Table). New MOs were demonstrated to target their intended sequence using EGFP reporter constructs engineered to contain target sequences (S10 Fig). MO-*csf3r*^ATG^ specificity was controlled by MO-*csf3r*^splice^; only MO-*csf3r*^ATG^ data are presented. MO-*il6ra*^ATG^ and MO-g*p130*^ATG^ served as specificity controls for each other as they targeted separate components of the same heterodimeric receptor complex.

### Inducible leukocyte ablation

*Tg(mpeg1*:*Gal4FF/UAS-E1b*:*Eco*.*nfsB-mCherry)* or *Tg(mpx*:*Kal4TA4/UAS-E1b*:*Eco*.*nfsB-mCherry)* embryos generated by intercrossing were treated with 10 mM metronidazole (Sigma M3761) from 28–52 hpf. The efficiency of macrophage ablation assessed using live imaging (S4 Movie) and LU at 24 h after treatment was ~70% (S9A Fig), comparable with the experience of others [25]. The efficiency of neutrophil ablation at 24 h after treatment was ~60% of Sudan Black positive neutrophils (S9B and S9C Fig). In infection experiments, metronidazole treatment was continued throughout the infection time course to restrict leukocyte recovery.

### Csf3b overexpression

Capped *csf3b* mRNA was transcribed from pCS2+ plasmids containing the cDNA [67] linearized by Not1-HF using the mMessage mMACHINE kit followed by RNA cleanup using RNeasy Mini Kit (Qiagen) according to manufacturer’s instructions. 5 μL was subjected to RNA electrophoresis for a quality check and stock aliquoted and stored at -70°C. 500–1000 pg of capped RNA was microinjected directly into the cell of 1-cell embryos. Control embryos received diluent alone.

### Mammalian macrophage infection with *Talaromyces* *marneffei*

J774 murine macrophages were seeded at a concentration of 1x10^5^ conidia/mL into a 6 well microtitre tray containing sterile coverslips and 2 mL of DMEM medium. Macrophages were incubated at 33°C or 37°C for 24 hours followed by the addition of 0.1 μg/mL lipopolysaccharide (LPS) and incubation for a further 24 hours. The cells were washed in PBS and 2 mL of complete DMEM medium containing 1x10^6^ conidia was added. A control lacking conidia was also performed. Macrophages were incubated for 2 hours at 37°C to allow conidia to be engulfed, washed once in PBS to remove non-phagocytosed conidia and incubated a further 24 hours at 33°C or 37°C. Macrophages were fixed in 4% paraformaldehyde and stained with 1 mg/mL fluorescent brightener 28 (calcofluor) to observe fungal cell walls. Mounted coverslips were examined using differential interference contrast and epifluorescence optics for cell wall staining and imaged on an Olympus IX70 microscope.

### Histology and stains

Standard 4% paraformaldehyde-fixed, paraffin-embedded sections were stained by hematoxylin and eosin or Grocott methanamine silver stains by the Walter and Eliza Hall Institute of Medical Research Histology Department. FACS-sorted leukocytes from infected *Tg(mpx*:*EGFP)* embryos were collected based on EGFP fluorescence and cytospun preparations stained with Grocott methanamine silver / Nuclear Fast Red.

### Microscopy and image processing

Routine brightfield and fluorescence imaging used a Zeiss Lumar V12 stereo dissecting microscope with an AxioCam MRm camera running AxioVision 4.8 software. Images were 1388x1040 pixels. Compound microscopy used an upright Nikon Optiphot-2 microscope with 40x and 100x objectives and a Zeiss AxioCam MRc5 Camera running AxioVision AC (Release 4.5) software. Images were 1292x968 pixels. Confocal microscopy used a Zeiss LSM 5 Live with a Plan-Apochromat 20x, 0.8 NA objective. Software was Zen (Version 4.0). Images were 16-bit 512 x 512 pixels. Z-depth ranged from 0–90 slices with Z-intervals optimized for 1:1:1 X:Y:Z reconstruction. Time intervals were optimized for each experiment and ranged between 30–200 s. Excitatory laser wavelengths were 405 nm for calcofluor, 489 nm for EGFP and 561 nm for mCherry. Emission detection used a BP495-555 filter for calcofluor and EGFP emission and a LP575 filter for mCherry emission.

Image processing was performed using Fiji (ImageJA 1.45b) and Imaris v5 (Bitplane). Colocalization analysis was performed using the Coloc function in Imaris, with thresholds set such that internal colocalization of each channel corresponded to the cell-specific fluorescent signal. Figures were constructed using Adobe CS5 Photoshop and Illustrator.

### Statistics

Descriptive and analytical statistics were prepared in Prism 5.0c (GraphPad Software Inc). Unless otherwise stated, data are mean±SEM, with p-values generated from two-tailed unpaired t-tests.

## Supporting information

S1 Table Antisense morpholino oligonucleotides used in this study. (DOCX)

S1 Fig Additional features of zebrafish *T*. *marneffei* infection at 28°C. (A-C) Maximum intensity projection confocal fluorescence z-stacks depicting stages of acuD:RFP *T*. *marneffei* infection in *Tg(mpx*:*EGFP)* zebrafish, allowing observation of fluorescent leukocytes, with calcofluor pre-staining allowing visualization of fungal conidia and RFP expression demonstrating their germination. Scale bars: 20 μm. dpi, days post infection.(A) Germination of conidia at 1 dpi with extension of RFP-positive germ tubes from extracellular calcofluor-stained conidia (arrowhead) adjacent to *Tg(mpx*:*EGFP)* leukocytes.(B) Destruction of fungal cells identified by RFP-positive debris within a neutrophil vacuole at 2 dpi (boxed).(C) Filamentous fungal cell growth (arrowhead) stretches and ruptures some leukocytes at 2 dpi.(D-F) Histology of infected zebrafish at different stages of *T*. *marneffei* infection. Fungal cells are stained black with Grocott’s methenamine silver stain against an Evan’s Blue counterstain (i, left panels). Tissues are visualized by hematoxylin and eosin staining of adjacent sections (ii, right panels). Scale bars: 20 μm.(D) Early granuloma formation at 2 dpi with accumulation of leukocytes around an infection focus (red arrowhead).(E) Infection focus in the brain at 3 dpi (red arrowhead).(F) Organising granuloma at 4 dpi, with epithelioid leukocytes surrounding a necrotic centre containing fungal debris (lower red arrowhead). Infected leukocyte nearby (upper red arrowhead) suggests dissemination of infection from the granuloma by leukocytes.(G) Low-power fluorescence image superimposed on brightfield image, showing granuloma formation at 4 dpi in tissue adjacent to the caudal hematopoietic tissue (CHT). Fluorescent *Tg(mpx*:*EGFP)* leukocytes (white arrowhead) have accumulated around a focus of germinated acuD:RFP *T*. *marneffei* cells (red arrowhead).(H) Maximum intensity projection of confocal z-stack showing accumulation of EGFP positive leukocytes around a focus of RFP-expressing germinated *T*. *marneffei*. Invasive filamentous growth (red arrowhead) radiates from the margins of the granuloma centre (white arrowhead). Scale bar: 20 μm.
(TIF)

S2 Fig Leukocyte migration and phagocytosis in *Aspergillus fumigatus* infection at 28°C or 33°C. (A) Numbers of migrating neutrophils (upper row) and macrophages (lower row) arrived at a local intramuscular site of live or dead *A*. *fumigatus* conidial microinjection.(B) Numbers of arrived neutrophils (upper row) and macrophages (lower row) that had phagocytosed fungal spores after local intramuscular inoculation of live or dead *A*. *fumigatus* conidia. Different coloured lines represent data followed longitudinally in n = 8–10 embryos per group, each embryo was separately imaged. In each scenario, the same colour indicates neutrophil and macrophages in the same embryo. p-values compared the 180 min timepoint only, using an unpaired 2-tailed t-test and the Bonferroni-Dunn correction for multiple comparisons. # indicates an embryo with a censored 180 min result, due to movement out of the imaged volume during microscopy.
(TIF)

S3 Fig Myelopoietic response to intravenous *Aspergillus fumigatus* infection at 28°C or 33°C. (A-B) Numbers of neutrophils (A) and macrophages (B) over a 4-day period following intravenous infection with live (left panels) and dead (right panels) *A*. *fumigatus* conidia.Different coloured dots represent embryos followed longitudinally in 3 independent experiments (n = 10/group in each experiment); superimposed in black are means±SD. P-values from unpaired 2-tailed t-test on pooled data and the Bonferroni-Dunn correction for multiple comparisons; for all other groups, p>0.05. hpi, hours post infection; dpi, days post infection; cnt, control (uninfected); inf, infected.
(TIF)

S4 Fig Impact of varying leukocyte populations on *Aspergillus fumigatus* germination at 28°C and 33°C. (A) Distribution of fungal form in morpholino-treated embryos with perturbed leukocyte specification at 24 hours post infection (hpi) at 28°C and 33°C. Form was assigned morphologically into 3 categories: ungerminated conidia, germling, or hyphal form.(B-C) Assessment of initial phagocytosis of *A*. *fumigatus* conidia following intravascular delivery. (B) Graph shows the total number of conidia, macrophages and neutrophils counted within the CHT at 2 hpi, and the subset of macrophages and neutrophils that contained conidia. (C) Scatterplot of leukocytes containing conidia versus the number of conidia demonstrates that the macrophage predominance in phagocytosis was independent of the number of conidia delivered. N = 42 embryos scored.n-values pooled from ≥ 3 independent experiments. In (A), for the 5 comparisons between the two temperatures, p>0.05 by Fisher’s Exact Test.
(TIF)

S5 Fig *T*. *marneffei* form in mammalian macrophages at 33°C or 37°C *in vitro*. (A) Appearance of *T*. *marneffei* conidia in J774 murine macrophages following 2 h of incubation at 37°C to permit engulfment. All phagocytosed, non-germinated conidia retain their original round/spherical form.(B) Appearance of engulfed *T*. *marneffei* conidia in J774 murine macrophages after 24 h of further incubation at either 33°C or 37°C. Elongated, oval yeast forms, including some with the characteristic medial septum of dividing yeast, are evident at both temperatures.(C) Quantification of proportion of *T*. *marneffei* at 26 hpi displaying oval or medially-septate yeast morphology at the two temperatures. Data are mean± SEM.
Scale bars: 10 μm.
(TIF)

S6 Fig Perturbation of leukocyte lineages skews phagocytosis following intravascular injection. (A) Measurements of cell volume for neutrophils (green) and macrophages (red) taken from confocal z-stacks of the caudal hematopoietic tissue (CHT) region in morphant embryos.(B) Measurement of fluorescence colocalization between calcofluor-labelled conidia and neutrophils (green) or macrophages (red) in the CHT at 2 hpi following vascular delivery.
N = 5 embryos analysed per condition collated from ≥ 3 experiments.
(TIF)

S7 Fig Expansion of neutrophils by *csf3b* overexpression does not enhance fungal clearance. (A) Overexpression of *csf3b* by mRNA microinjection results in pronounced expansion of neutrophils (ii) and mild expansion of macrophages (iv) compared to their respective diluent-injected controls (i and iii).(B) Overexpression of *csf3b* does not affect *T*. *marneffei* CFU counts at 24 hpi compared to controls. Black and red points represent results obtained from independent experiments.
(TIF)

S8 Fig The role of myeloperoxidase during *T*. *marneffei* infection at 33°C. (A) Comparative CFU time-course of wildtype and Mpx-deficient embryos. No significant difference was observed between groups. Data are mean±SEM from 3 independent experiments with 5 embryos (pooled)/group/timepoint/experiment. P-values from unpaired two-tailed t-test.(B) Neutrophil populations during experiments shown in (A). An augmented neutrophil response occurred in Mpx-deficient embryos at 3 dpi. Data are mean±SEM from 3 independent experiments. N = 5 embryos/group/timepoint. P-values from unpaired two-tailed t-test comparing wildtype and *mpx*^-/-^ groups.
(TIF)

S9 Fig Quantification of nitroreductase-mediated leukocyte ablation. (A) Graph shows quantification of leukocyte units (LUs) for macrophages (NTR-mCherry, red bars) and neutrophils (GFP, green bars) in compound *Tg(mpeg*:*Gal4/UAS*:*nfsB-mCherry/mpx*:*EGFP)* transgenic zebrafish embryos treated with DMSO or Metronidazole. A significant reduction in NTR-expressing macrophages, but not neutrophils was observed in Metronidazole-treated embryos compared to DMSO-treated controls. Data are mean±SEM, n≥5 embryos/group/experiment, n≥3 experiments. Statistics: two-tailed t-test.(B) Counts of mCherry-positive cells in the tail region of *Tg(mpx*:*KalTA4/UAS*:*nfsB-mCherry)* transgenic embryos following treatment with DMSO or Metronidazole. A significant reduction in the number of mCherry-positive cells was observed. Data are mean±SD, n = 15 embryos/group pooled from N = 2 experiments. Statistics: two-tailed t-test.(C) Additional evidence for successful neutrophil ablation by Sudan Black B staining of embryos following DMSO (i) or Metronidazole (ii) treatment. Counts of Sudan Black positive cells in the tail region (iii) confirmed a significant reduction in Metronidazole-treated embryos. Data are mean±SD. N = 8 embryos from N = 1 experiment. Statistics: two-tailed t-test.
(TIF)

S10 Fig Demonstration of morpholino oligonucleotide on-target action. (A-C) Images show abrogation of expression from MO-EGFP mRNA (EGFP fused in-frame to an engineered 5' ATG sequence intended to be targeted by the respective antisense morpholino oligonucleotide (MO) being tested) following MO treatment compared to untreated and EGFP mRNA-only controls, demonstrating on-target MO knockdown capability. MO delivery is traced by co-injection with Rhodamine dextran (Rhod).(D) Physiological specificity of the ATG-targeting *csf3r*-MO is confirmed by the concordant neutrophil-depletion phenotypes 4 dpi following knockdown of *csf3r* by either the ATG-MO (1) or splice-targeting morpholino (2). Data are mean+SD, n = 10 embryos/group, p-values from unpaired two-tailed t-test.
(TIF)

S1 Movie Phagocytosis of a calcofluor-stained *T*. *marneffei* conidium by a mCherry labelled macrophage following inoculation into the somite of a *Tg(mpeg1*:*mCherry)* zebrafish embryo. (AVI)

S2 Movie Phagocytosis of a calcofluor-stained *T*. *marneffei* conidium by a EGFP labelled neutrophil following inoculation into the somite of a *Tg(mpx*:*EGFP)* zebrafish embryo. (AVI)

S3 Movie Migration and phagocytic response of neutrophils (green, EGFP-CAAX) and macrophages (red, mCherry-CAAX) in response to somite inoculation of calcofluor-labelled *T*. *marneffei* conidia (blue). (AVI)

S4 Movie Live-imaging of macrophage ablation following metronidazole treatment of a compound transgenic *Tg(mpeg1*:*Gal4FF/UAS- E1b*:*Eco*.*nfsB-mCherry/UAS*:*Kaede)* embryo. (AVI)
