# Supplementary material for: Macrophages protect Talaromyces marneffei conidia from myeloperoxidase-dependent neutrophil fungicidal activity during infection establishment in vivo
Source: PLoS Pathog. 2018 Jun 8;14(6):e1007063. doi: 10.1371/journal.ppat.1007063 (PMC6010348; doi:10.1371/journal.ppat.1007063)
Supplement: S1 Table — (DOCX) [file ppat.1007063.s001.docx]

**Table S1: Antisense morpholino oligonucleotides**

| **Morpholino** | **Sequence** | **Purpose** | **Reference** |
| --- | --- | --- | --- |
| MO-Control | 5'-CCTCTTACCTCAGTTACAATTTATA-3' | Acts as a negative control for off-target MO toxicity | GeneTools (Eugene, OR) |
| MO-*csf3r*^ATG^ | 5'-GAAGCACAAGCGAGACGGATGCCAT-3' | Results in neutrophil depletion due to knockdown of Csf3-receptor signaling | (Liongue et al., 2009) |
| MO-*csf3r*^splice^ | 5'-AAGCAAAACCGTGTTACCATTTCAGA-3' | Acts as a specificity control for MO-*csf3r*^ATG^ by targeting splice-site | New |
| MO-*spi1* | 5'-GATATACTGATACTCCATTGGTGGT-3' | Impairs primitive myelopoiesis by knockdown of the master transcription factor Spi1 | (Rhodes et al., 2005) |
| MO*-irf8* | 5'-TCAGTCTGCGACCGCCCGAGTTCAT-3' | Skews leukocyte specification towards granulocyte lineage at expense of macrophages by knockdown of the transcription factor Irf8. | (Li et al., 2010) |
| MO-*gp130* | 5'-ACAGCCAATGATGTGAAGTGTCCAT-3' | Knockdown of receptor signaling through heterodimeric receptor complexes containing Gp130 | New |
| MO-*il6ra* | 5'-GTCTAGTGAATCCAAAACCCTCCAT-3' | Knockdown of heterodimeric interleukin 6-receptor signaling by alpha chain depletion | New |

**References**

Li, L., Jin, H., Xu, J., Shi, Y., and Wen, Z. (2010). Irf8 regulates macrophage versus neutrophil fate during zebrafish primitive myelopoiesis. Blood.

Liongue, C., Hall, C.J., O'Connell, B.A., Crosier, P., and Ward, A.C. (2009). Zebrafish granulocyte colony-stimulating factor receptor signaling promotes myelopoiesis and myeloid cell migration. Blood *113*, 2535-2546.

Rhodes, J., Hagen, A., Hsu, K., Deng, M., Liu, T.X., Look, A.T., and Kanki, J.P. (2005). Interplay of pu.1 and gata1 determines myelo-erythroid progenitor cell fate in zebrafish. Dev Cell *8*, 97-108.
