# Supplementary figures and images for: Macrophages protect Talaromyces marneffei conidia from myeloperoxidase-dependent neutrophil fungicidal activity during infection establishment in vivo
Source: PLoS Pathog. 2018 Jun 8;14(6):e1007063. doi: 10.1371/journal.ppat.1007063 (PMC6010348; doi:10.1371/journal.ppat.1007063)

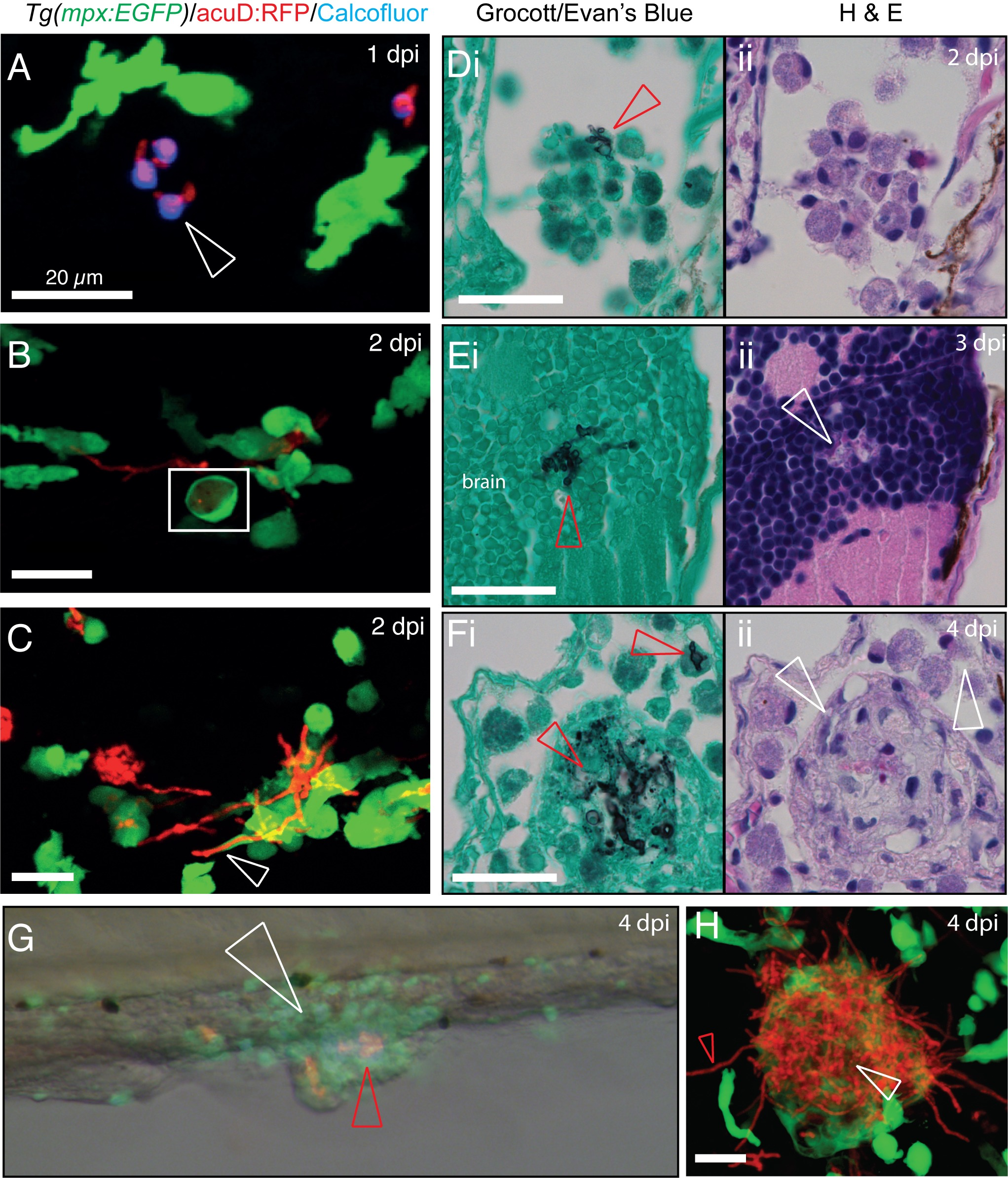

Supplement: S1 Fig — (A-C) Maximum intensity projection confocal fluorescence z-stacks depicting stages of acuD:RFP T. marneffei infection in Tg(mpx:EGFP) zebrafish, allowing observation of fluorescent leukocytes, with calcofluor pre-staining allowing visualization of fungal conidia and RFP expression demonstrating their germination. Scale bars: 20 μm. dpi, days post infection.(A) Germination of conidia at 1 dpi with extension of RFP-positive germ tubes from extracellular calcofluor-stained conidia (arrowhead) adjacent to Tg(mpx:EGFP) leukocytes.(B) Destruction of fungal cells identified by RFP-positive debris within a neutrophil vacuole at 2 dpi (boxed).(C) Filamentous fungal cell growth (arrowhead) stretches and ruptures some leukocytes at 2 dpi.(D-F) Histology of infected zebrafish at different stages of T. marneffei infection. Fungal cells are stained black with Grocott’s methenamine silver stain against an Evan’s Blue counterstain (i, left panels). Tissues are visualized by hematoxylin and eosin staining of adjacent sections (ii, right panels). Scale bars: 20 μm.(D) Early granuloma formation at 2 dpi with accumulation of leukocytes around an infection focus (red arrowhead).(E) Infection focus in the brain at 3 dpi (red arrowhead).(F) Organising granuloma at 4 dpi, with epithelioid leukocytes surrounding a necrotic centre containing fungal debris (lower red arrowhead). Infected leukocyte nearby (upper red arrowhead) suggests dissemination of infection from the granuloma by leukocytes.(G) Low-power fluorescence image superimposed on brightfield image, showing granuloma formation at 4 dpi in tissue adjacent to the caudal hematopoietic tissue (CHT). Fluorescent Tg(mpx:EGFP) leukocytes (white arrowhead) have accumulated around a focus of germinated acuD:RFP T. marneffei cells (red arrowhead).(H) Maximum intensity projection of confocal z-stack showing accumulation of EGFP positive leukocytes around a focus of RFP-expressing germinated T. marneffei. Invasive filamentous growth (red arrow [file ppat.1007063.s002.tif]

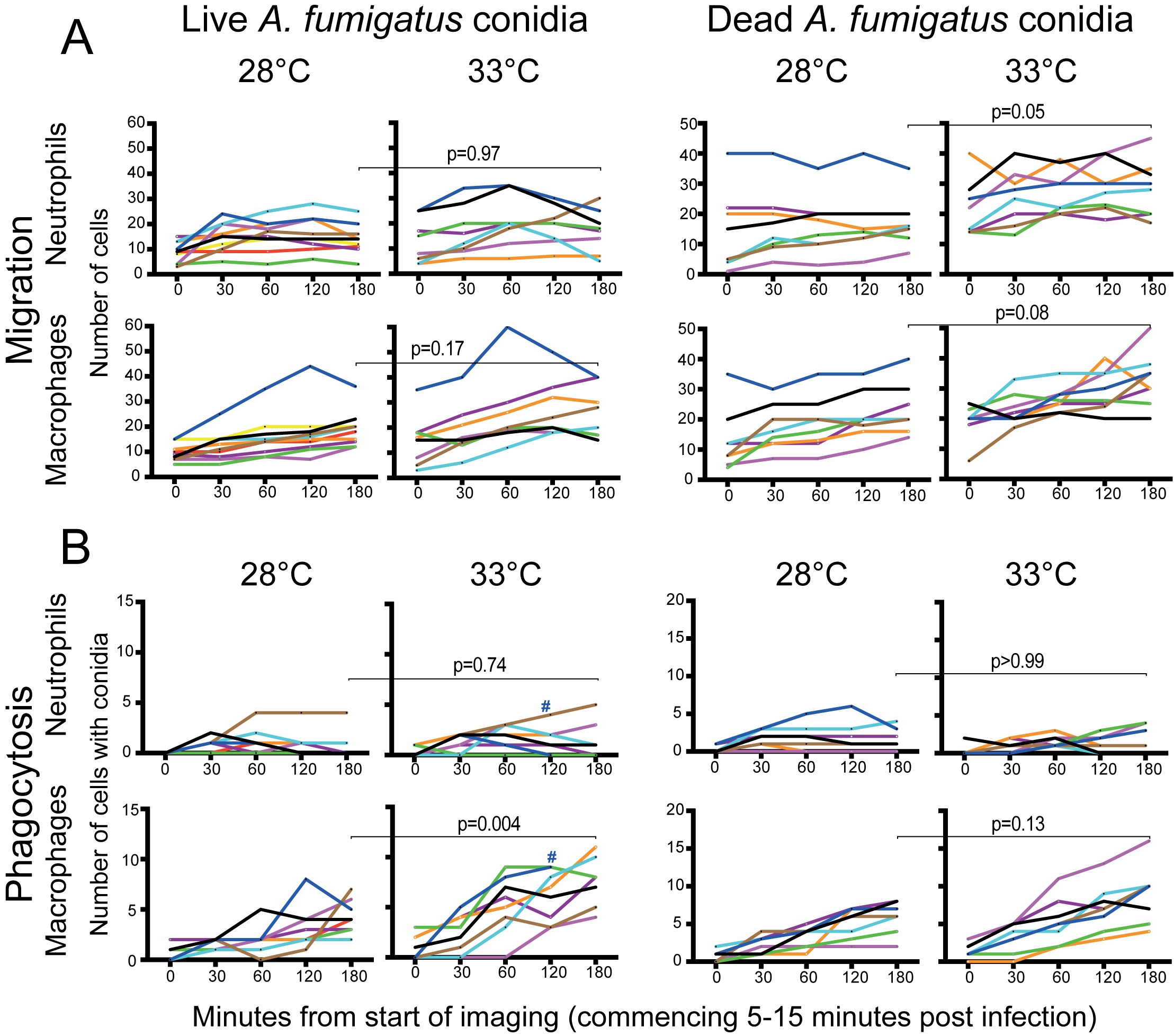

Supplement: S2 Fig — (A) Numbers of migrating neutrophils (upper row) and macrophages (lower row) arrived at a local intramuscular site of live or dead A. fumigatus conidial microinjection.(B) Numbers of arrived neutrophils (upper row) and macrophages (lower row) that had phagocytosed fungal spores after local intramuscular inoculation of live or dead A. fumigatus conidia. Different coloured lines represent data followed longitudinally in n = 8–10 embryos per group, each embryo was separately imaged. In each scenario, the same colour indicates neutrophil and macrophages in the same embryo. p-values compared the 180 min timepoint only, using an unpaired 2-tailed t-test and the Bonferroni-Dunn correction for multiple comparisons. # indicates an embryo with a censored 180 min result, due to movement out of the imaged volume during microscopy. (TIF) [file ppat.1007063.s003.tif]

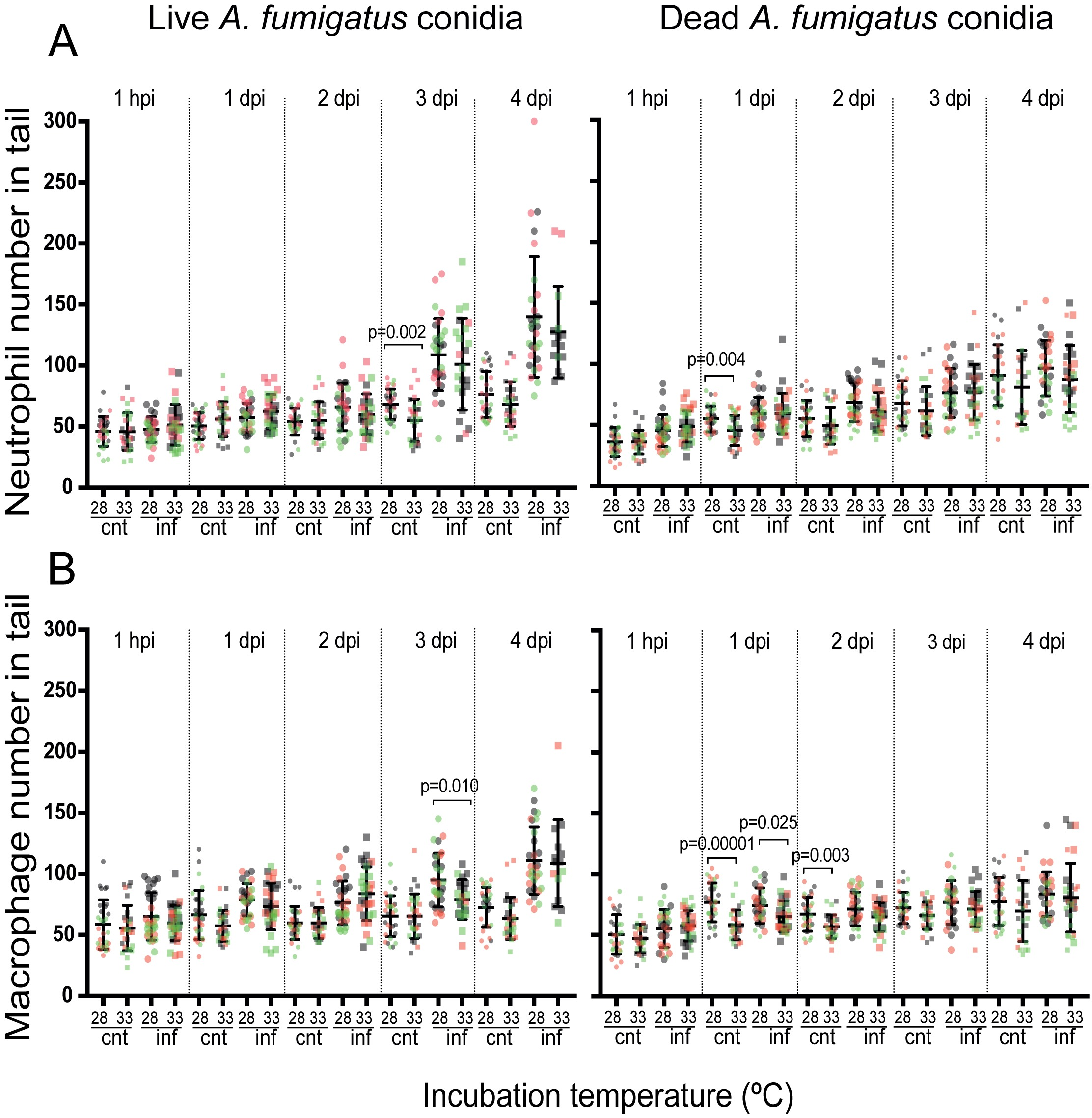

Supplement: S3 Fig — (A-B) Numbers of neutrophils (A) and macrophages (B) over a 4-day period following intravenous infection with live (left panels) and dead (right panels) A. fumigatus conidia.Different coloured dots represent embryos followed longitudinally in 3 independent experiments (n = 10/group in each experiment); superimposed in black are means±SD. P-values from unpaired 2-tailed t-test on pooled data and the Bonferroni-Dunn correction for multiple comparisons; for all other groups, p>0.05. hpi, hours post infection; dpi, days post infection; cnt, control (uninfected); inf, infected. (TIF) [file ppat.1007063.s004.tif]

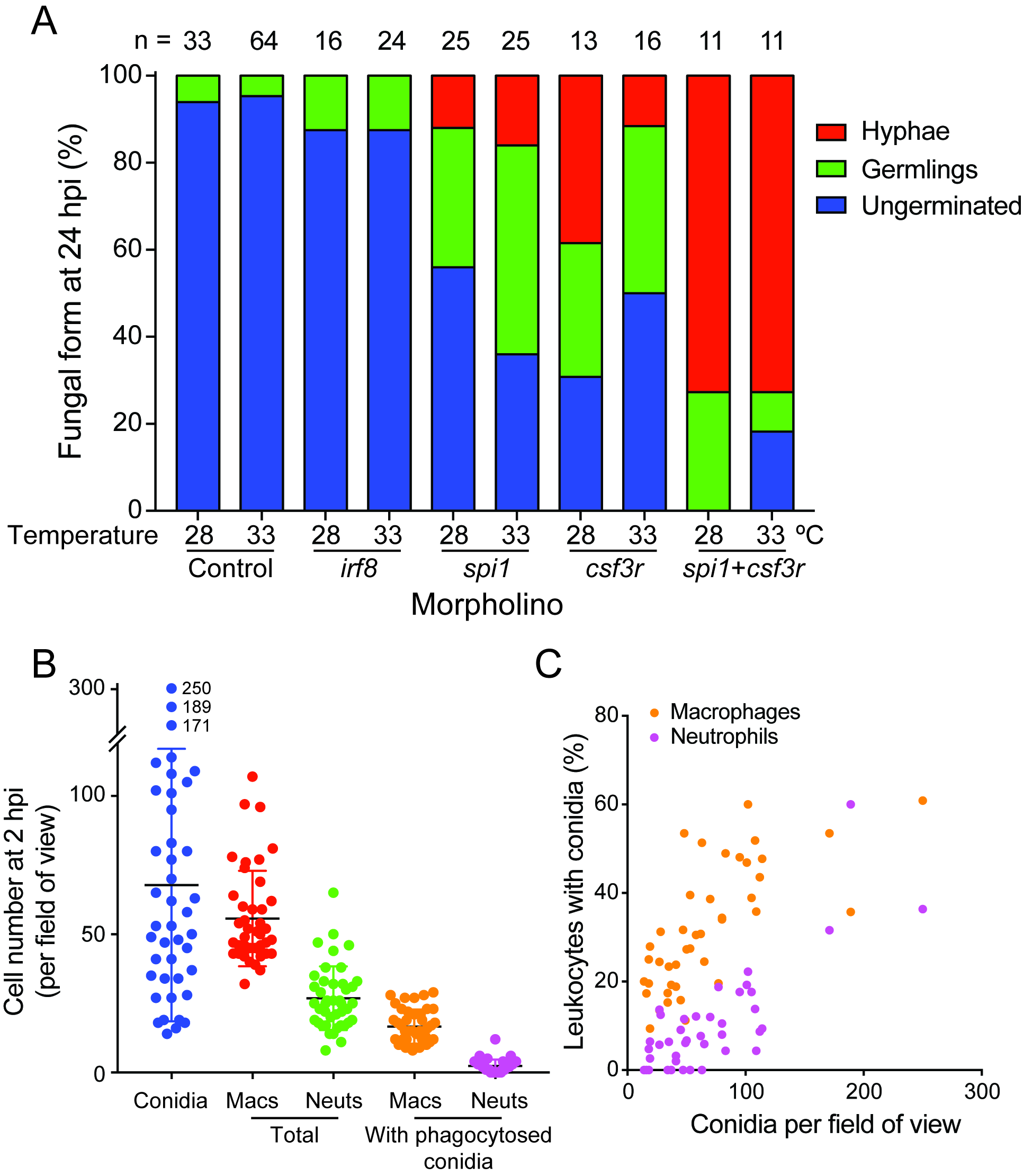

Supplement: S4 Fig — (A) Distribution of fungal form in morpholino-treated embryos with perturbed leukocyte specification at 24 hours post infection (hpi) at 28°C and 33°C. Form was assigned morphologically into 3 categories: ungerminated conidia, germling, or hyphal form.(B-C) Assessment of initial phagocytosis of A. fumigatus conidia following intravascular delivery. (B) Graph shows the total number of conidia, macrophages and neutrophils counted within the CHT at 2 hpi, and the subset of macrophages and neutrophils that contained conidia. (C) Scatterplot of leukocytes containing conidia versus the number of conidia demonstrates that the macrophage predominance in phagocytosis was independent of the number of conidia delivered. N = 42 embryos scored.n-values pooled from ≥ 3 independent experiments. In (A), for the 5 comparisons between the two temperatures, p>0.05 by Fisher’s Exact Test. (TIF) [file ppat.1007063.s005.tif]

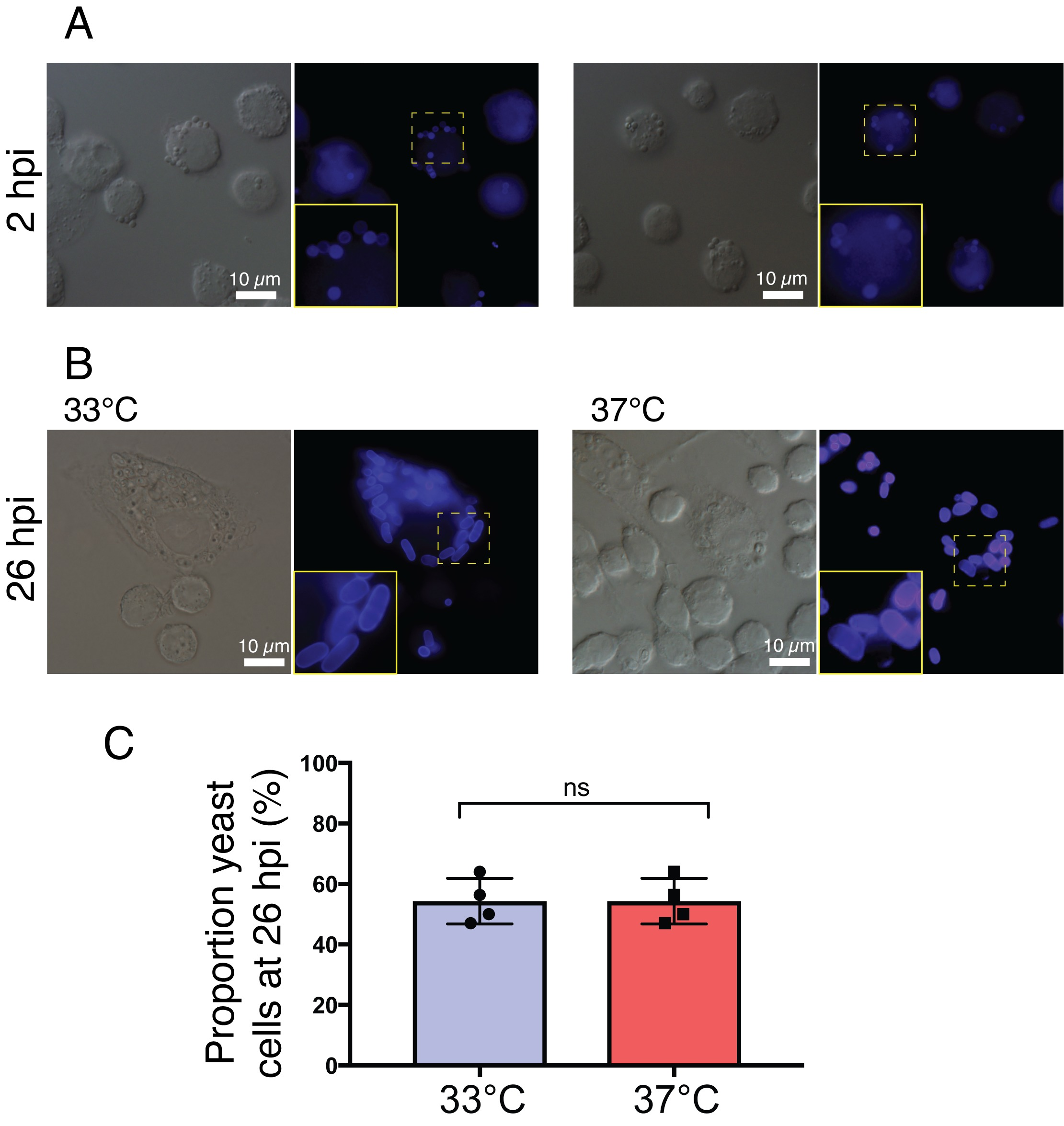

Supplement: S5 Fig — (A) Appearance of T. marneffei conidia in J774 murine macrophages following 2 h of incubation at 37°C to permit engulfment. All phagocytosed, non-germinated conidia retain their original round/spherical form.(B) Appearance of engulfed T. marneffei conidia in J774 murine macrophages after 24 h of further incubation at either 33°C or 37°C. Elongated, oval yeast forms, including some with the characteristic medial septum of dividing yeast, are evident at both temperatures.(C) Quantification of proportion of T. marneffei at 26 hpi displaying oval or medially-septate yeast morphology at the two temperatures. Data are mean± SEM. Scale bars: 10 μm. (TIF) [file ppat.1007063.s006.tif]

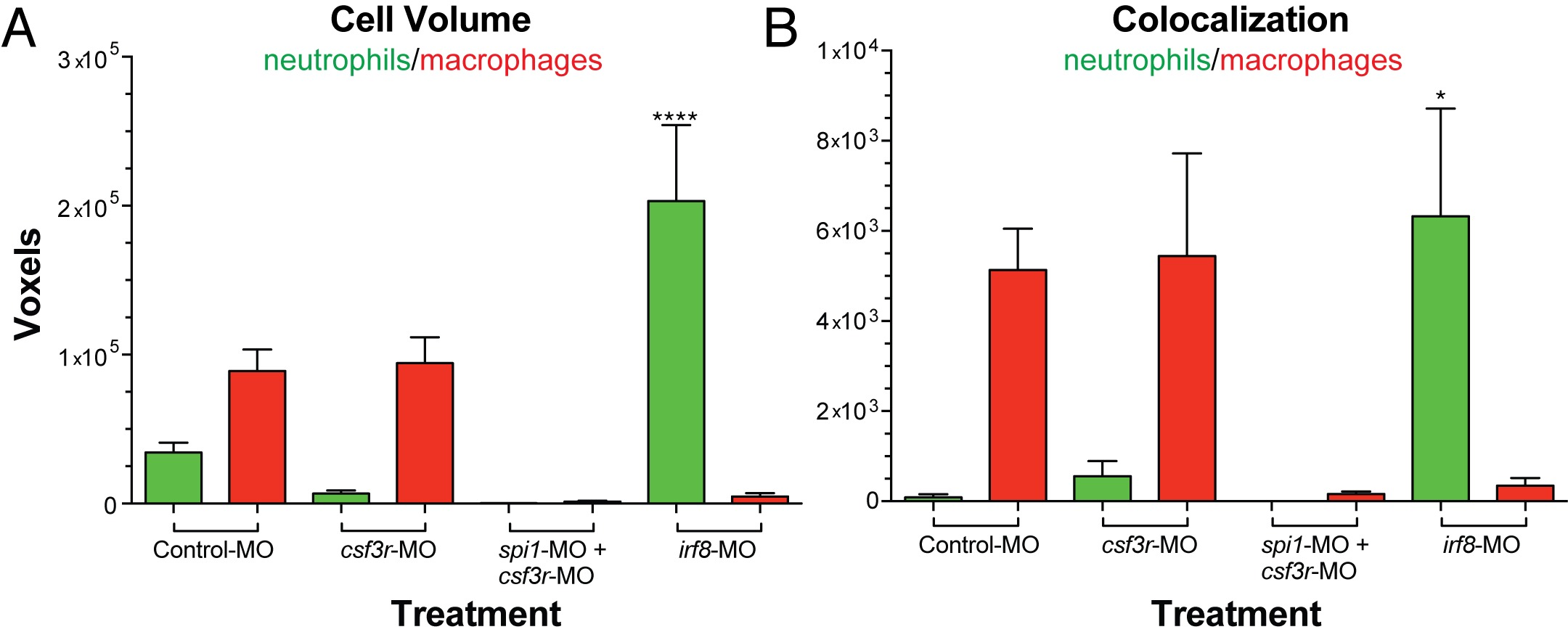

Supplement: S6 Fig — (A) Measurements of cell volume for neutrophils (green) and macrophages (red) taken from confocal z-stacks of the caudal hematopoietic tissue (CHT) region in morphant embryos.(B) Measurement of fluorescence colocalization between calcofluor-labelled conidia and neutrophils (green) or macrophages (red) in the CHT at 2 hpi following vascular delivery. N = 5 embryos analysed per condition collated from ≥ 3 experiments. (TIF) [file ppat.1007063.s007.tif]

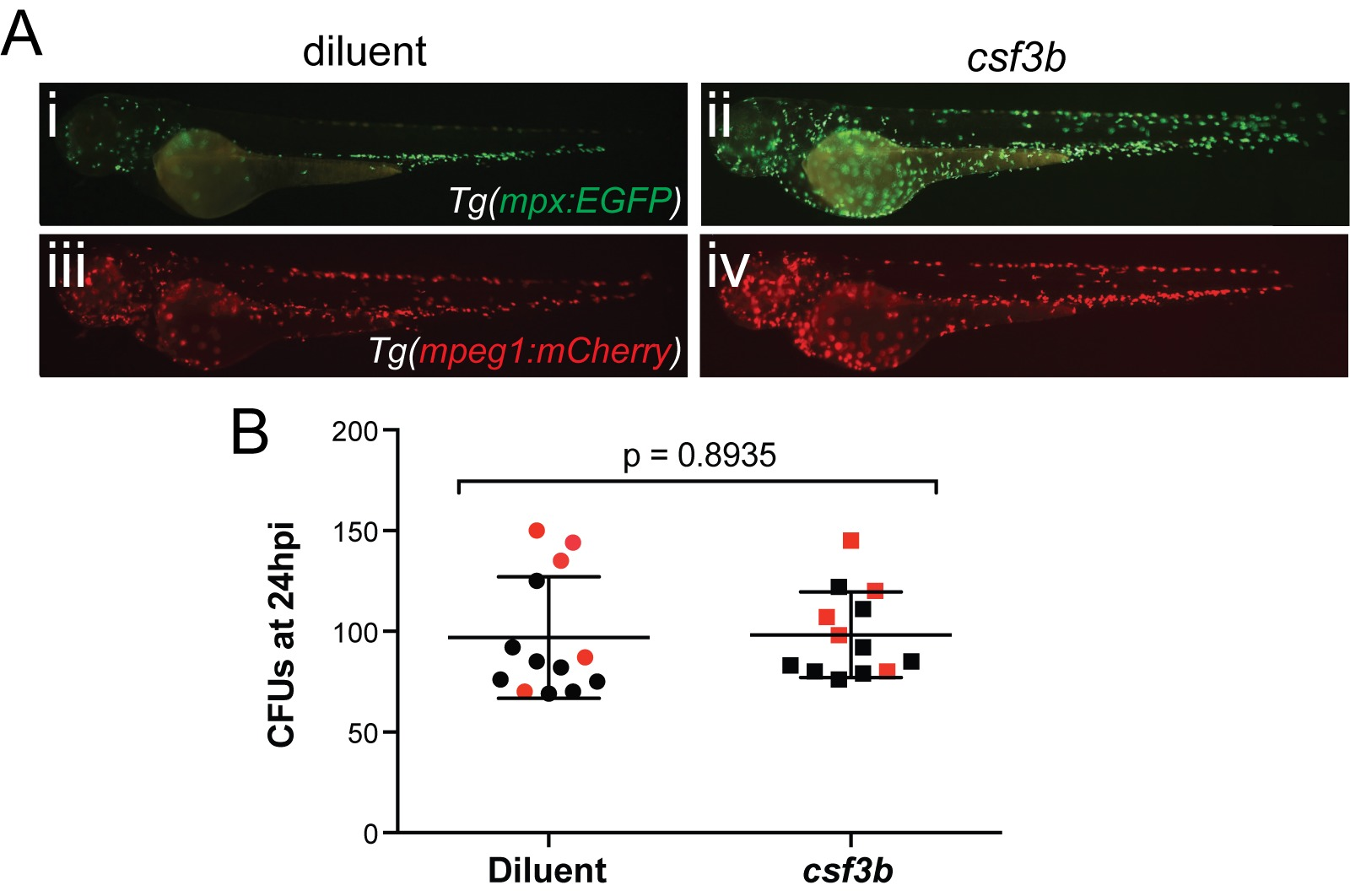

Supplement: S7 Fig — (A) Overexpression of csf3b by mRNA microinjection results in pronounced expansion of neutrophils (ii) and mild expansion of macrophages (iv) compared to their respective diluent-injected controls (i and iii).(B) Overexpression of csf3b does not affect T. marneffei CFU counts at 24 hpi compared to controls. Black and red points represent results obtained from independent experiments. (TIF) [file ppat.1007063.s008.tif]

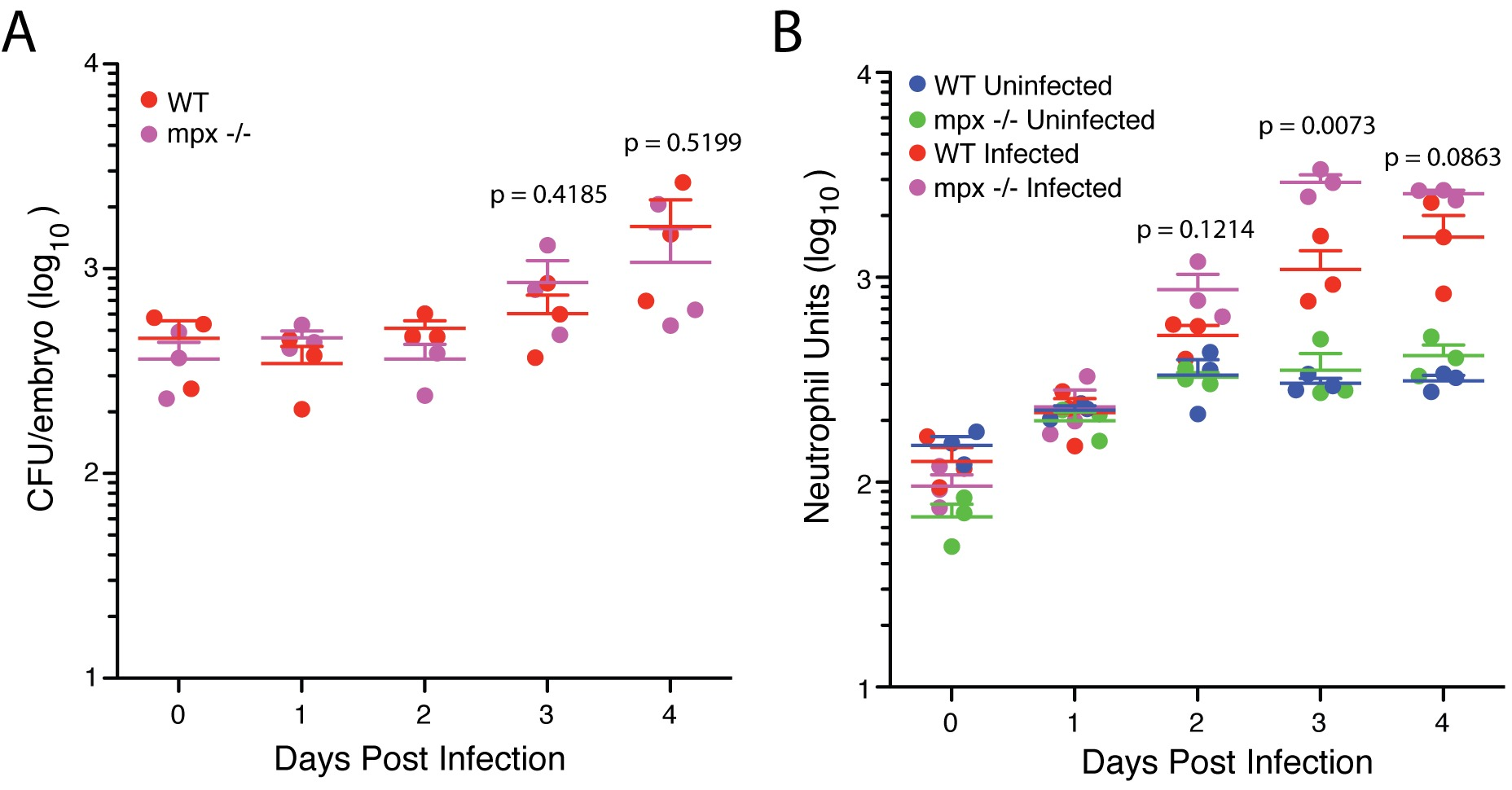

Supplement: S8 Fig — (A) Comparative CFU time-course of wildtype and Mpx-deficient embryos. No significant difference was observed between groups. Data are mean±SEM from 3 independent experiments with 5 embryos (pooled)/group/timepoint/experiment. P-values from unpaired two-tailed t-test.(B) Neutrophil populations during experiments shown in (A). An augmented neutrophil response occurred in Mpx-deficient embryos at 3 dpi. Data are mean±SEM from 3 independent experiments. N = 5 embryos/group/timepoint. P-values from unpaired two-tailed t-test comparing wildtype and mpx-/- groups. (TIF) [file ppat.1007063.s009.tif]

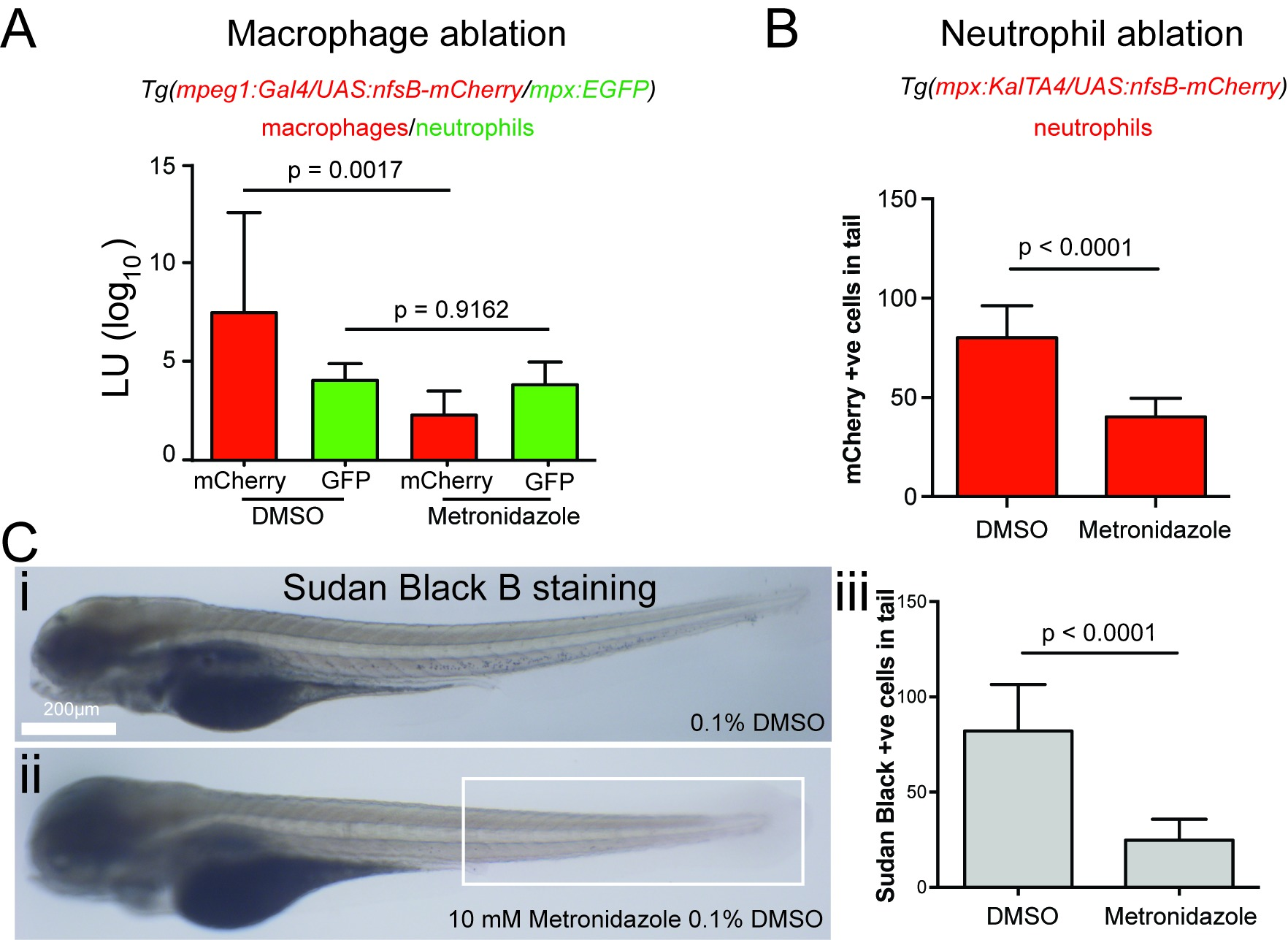

Supplement: S9 Fig — (A) Graph shows quantification of leukocyte units (LUs) for macrophages (NTR-mCherry, red bars) and neutrophils (GFP, green bars) in compound Tg(mpeg:Gal4/UAS:nfsB-mCherry/mpx:EGFP) transgenic zebrafish embryos treated with DMSO or Metronidazole. A significant reduction in NTR-expressing macrophages, but not neutrophils was observed in Metronidazole-treated embryos compared to DMSO-treated controls. Data are mean±SEM, n≥5 embryos/group/experiment, n≥3 experiments. Statistics: two-tailed t-test.(B) Counts of mCherry-positive cells in the tail region of Tg(mpx:KalTA4/UAS:nfsB-mCherry) transgenic embryos following treatment with DMSO or Metronidazole. A significant reduction in the number of mCherry-positive cells was observed. Data are mean±SD, n = 15 embryos/group pooled from N = 2 experiments. Statistics: two-tailed t-test.(C) Additional evidence for successful neutrophil ablation by Sudan Black B staining of embryos following DMSO (i) or Metronidazole (ii) treatment. Counts of Sudan Black positive cells in the tail region (iii) confirmed a significant reduction in Metronidazole-treated embryos. Data are mean±SD. N = 8 embryos from N = 1 experiment. Statistics: two-tailed t-test. (TIF) [file ppat.1007063.s010.tif]

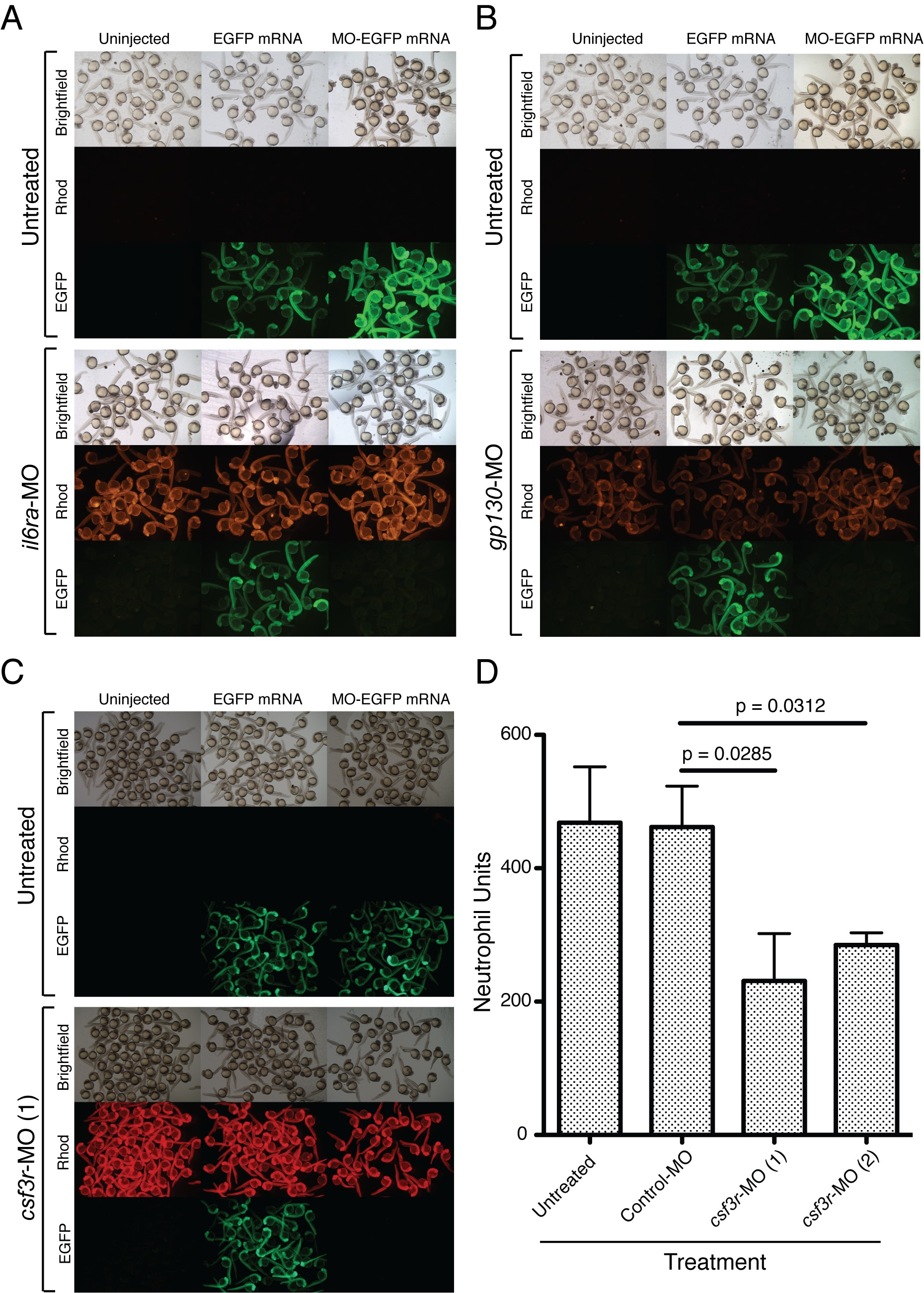

Supplement: S10 Fig — (A-C) Images show abrogation of expression from MO-EGFP mRNA (EGFP fused in-frame to an engineered 5' ATG sequence intended to be targeted by the respective antisense morpholino oligonucleotide (MO) being tested) following MO treatment compared to untreated and EGFP mRNA-only controls, demonstrating on-target MO knockdown capability. MO delivery is traced by co-injection with Rhodamine dextran (Rhod).(D) Physiological specificity of the ATG-targeting csf3r-MO is confirmed by the concordant neutrophil-depletion phenotypes 4 dpi following knockdown of csf3r by either the ATG-MO (1) or splice-targeting morpholino (2). Data are mean+SD, n = 10 embryos/group, p-values from unpaired two-tailed t-test. (TIF) [file ppat.1007063.s011.tif]
